# The role of East Asian monsoon system in shaping population divergence and dynamics of a constructive desert shrub *Reaumuria soongarica*

**DOI:** 10.1038/srep15823

**Published:** 2015-10-29

**Authors:** Hengxia Yin, Xia Yan, Yong Shi, Chaoju Qian, Zhonghu Li, Wen Zhang, Lirong Wang, Yi Li, Xiaoze Li, Guoxiong Chen, Xinrong Li, Eviatar Nevo, Xiao-Fei Ma

**Affiliations:** 1 Key Laboratory of Stress Physiology and Ecology in Cold and Arid Regions, Gansu Province, Department of Ecology and Agriculture Research, Cold and Arid Regions Environmental and Engineering Research Institute, Chinese Academy of Sciences, Lanzhou 730000, Gansu, China; 2 University of Chinese Academy of Sciences, Beijing 100049, China; 3 Key Laboratory of Eco-hydrology and of Inland River Basin, Cold and Arid Regions Environmental and Engineering Research Institute, Chinese Academy of Sciences, Lanzhou 730000, Gansu, China; 4 Northwest university, Xi’an 710075, Shanxi, China; 5 College of Forestry Science, Gansu Agricultural University, Lanzhou 730000, Gansu, China; 6 Key Lab of Desert and Desertification, Cold and Arid Regions Environmental and Engineering Research Institute of Chinese Academy of Sciences, Lanzhou 730000, Gansu, China; 7 Shapotou Desert Research and Experiment Station, Cold and Arid Regions Environmental and Engineering Research Institute, Chinese Academy of Sciences, Lanzhou 730000, Gansu, China; 8 Institute of Evolution, University of Haifa, Haifa 31905, Israel

## Abstract

Both of the uplift of Qinghai-Tibet Plateau (QTP) and the development of East Asian monsoon system (EAMS) could have comprehensively impacted the formation and evolution of Arid Central Asia (ACA). To understand how desert plants endemic to ACA responded to these two factors, we profiled the historical population dynamics and distribution range shift of a constructive desert shrub *Reaumuria soongarica* (Tamaricaceae) based on species wide investigation of sequence variation of chloroplast DNA and nuclear ribosomal ITS. Phylogenetic analysis uncovered a deep divergence occurring at ca. 2.96 Mya between the western and eastern lineages of *R. soongarica*, and ecological niche modeling analysis strongly supported that the monsoonal climate could have fragmented its habitats in both glacial and interglacial periods and impelled its intraspecific divergence. Additionally, the population from the east monsoonal zone expanded rapidly, suggesting that the local monsoonal climate significantly impacted its population dynamics. The isolation by distance tests supported strong maternal gene flow along the direction of the East Asian winter monsoon, whose intensification induced the genetic admixture along the latitudinal populations of *R. soongarica*. Our results presented a new case that the development of EAMS had prominently impacted the intraspecific divergence and population dynamics of this desert plant.

Global climate fluctuations and geological events were major forces in shaping the current vegetation distribution and geographical structure of genetic diversity of species in the Northern Hemisphere^1^. Exploring the patterns of species responding to climatic and geological events could help us understand how species’ distributions and their population dynamics are affected under climate scenarios^2^. Plenty of investigations focusing on the Qinghai-Tibetan Plateau (QTP) and adjacent regions have hypothesized that the QTP uplift might have triggered the evolutionary radiations of native species, meanwhile, global climate cyclic fluctuations during the Pleistocene could have also immensely influenced their distribution ranges and genetic patterns^3^,^4^. Up to date, however, the effects of geological and climatic scenarios on species diversification had not been well dissected in the organisms native to QTP^5^.

Arid Central Asia (also called arid Asian interior, ACA in this paper), adjoining to the QTP and accounting for one third of the arid regions in the world, had greatly attracted geological and ecological concerns with its formation and spatial and temporal evolution, especially the processes of aridification^6^. It was proposed that the aridification of ACA has been profoundly aggravated by two factors: the QTP uplifts and East Asian monsoon system (EAMS). The phased uplifts of QTP originated from the collision of the Indian subcontinent with Eurasia in the Eocene, extensive uplifts during ca. 15–13 Mya (million years ago), 8–7 Mya, and the last rapid uplift during 3.6–1.2 Mya^7^,^8^,^9^,^10^,^11^,^12^. The further aridification of ACA could be associated with the development of modern EAMS during the Pliocene and Pleistocene^7^,^8^,^13^,^14^, especially with the increasing variability and strengthening of East Asian winter monsoon (EAWM) since 2.6 Mya^7^. The aridification of ACA and the underlying monsoonal climate could have shaped the ranges and the present genetic constitution of the biotas in the region. For example, with the monsoonal climate oscillation during the Pleistocene, the eastern deserts (Tengger and Badain Jaran deserts) ranges have shifted more significantly than that of the western deserts (Taklimakan desert)^15^, and these changes could have drastically shifted the plant communities^14^. To date, a few studies have revealed that desert plants of ACA experienced episodes of population differentiation and expansion during their evolutionary histories^16^,^17^,^18^. Although the EAMS had been impacting the vegetation of ACA during late Pliocene and Pleistocene^7^, the role of EAMS in shaping species diversity and population dynamics has not been well addressed yet. Deeper investigation on evolutionary history on these desert plants could provide a platform to discuss how EAMS impacted their habitat fragmentations and intraspecific divergences, and could also acquire the information of aridification of ACA and the evolution of EAWM in the phylogeographic view.

*Reaumuria soongarica* (Tamaricaceae), a Tertiary relic shrub and typical xerophyte^19^, is widely distributed in all the desert regions across ACA^20^. As a constructive and zonal species, the plant plays a crucial role in sustaining fragile desert ecosystems, including Tengger Desert, Badain Jaran Desert, Gurbantunggut Desert, Gashun Gobi, Kumtag Desert, Qaidam Basin and Taklimakan Desert (www.eflora.org). The geographical distribution range of *R. soongarica* is covering the whole ACA and also *in situ* fragmented by the QTP, which could provide an ideal model for understanding the demographic history of desert plants in response to the uplift of QTP and monsoonal climate oscillation. Previous phylogeographical investigation on one chloroplast(cp) DNA fragment of *R. soongarica* has revealed a significant intraspecific divergence that occurred from 1.1 to 3.5 Mya between two regional groups adjoining to QTP^21^. However, partly due to the insufficient informative sites and incomplete sampling, it could not adequately dissect the effects of paleogeological and/or paleoclimatic changes on the demographic history of *R. soongarica*. Here, based on the species wide variations on five cpDNA fragments (maternally inherited)^22^,^23^,^24^ and one nuclear ribosomal internal transcribed spacer (nrITS, biparentally inherited) region from 34 populations across the ACA, we profiled the population structure and dynamics with the coalescent-based program MIGRATE-N^25^ and reconstructed its habitat fragmentation and range shift during Last Glacial Maximum (LGM) and Last Interglacial (LIG) periods with ecological niche modeling (ENM)^26^. We were aiming to elucidate the roles of the EAMS in shaping the evolutionary history of this desert plant by addressing the following three questions: 1) How the geological and monsoonal climatic change affected the intraspecific divergence of *R. soongarica*? 2) Whether the population dynamics among the regional groups were affected by local monsoonal or global climate change? 3) How the EAMS affected the gene flow among the regional groups of *R. soongarica*? This work would shed light on our understanding on the demographic history of constructive species in the fragile desert ecosystems in response to the monsoonal climate change, and also supported empirical phylogeographic data to understand the spatial and temporal evolution of ACA and EAMS.

## Results

### Genealogies and geographical distributions of haplotypes.

**Figure 1:**
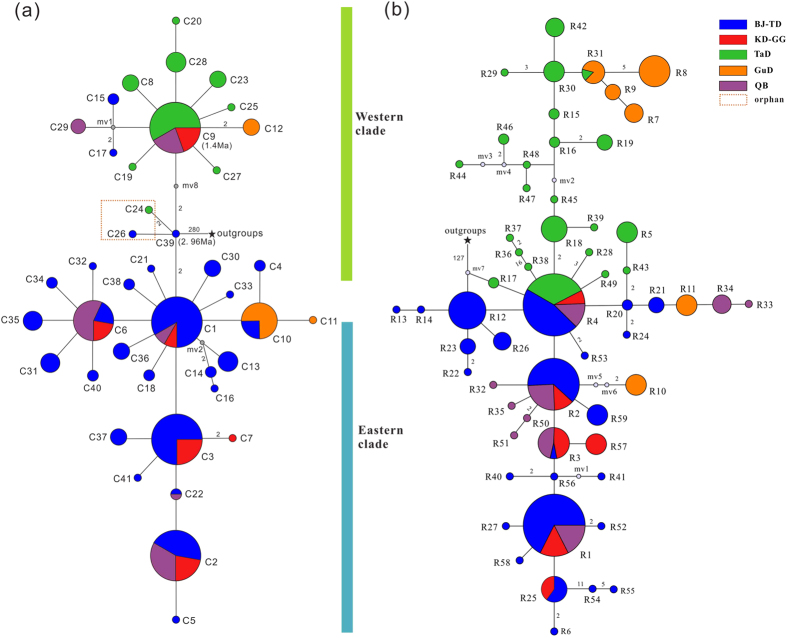
Median-joining networks of (**a**) 41 chlorotypes (C1-C41) and (**b**) 59 nuclear ribotypes (R1-R59) in *R. soongarica* from the five groups in ACA, e.g. Taklamakan Desert (TaD), Gurbantunggut Desert (GuD), Qaidam Basin (QB), Kumtag Desert and Gashun Gobi (KD-GG), and Badain Jaran and Tengger deserts (BJ-TD). The size of the circles corresponds to the frequency of each haplotype. Small gray circles indicate unsampled or extinct haplotypes. Each number near the line represents the mutational steps interconnecting two haplotypes, and only the steps over two mutations are listed. There were three mutation steps between C39 and C9, as each mutation represents 5.35 × 10^5^ year (1 substitution representing the divergence time of 1/[4.87 × 10^−10^ s/s/y × 3,836 bps) years]. Chlorotypes C39 and C9 are presented at ca. 2.96 and 1.4 Mya, respectively.

**Figure 2:**
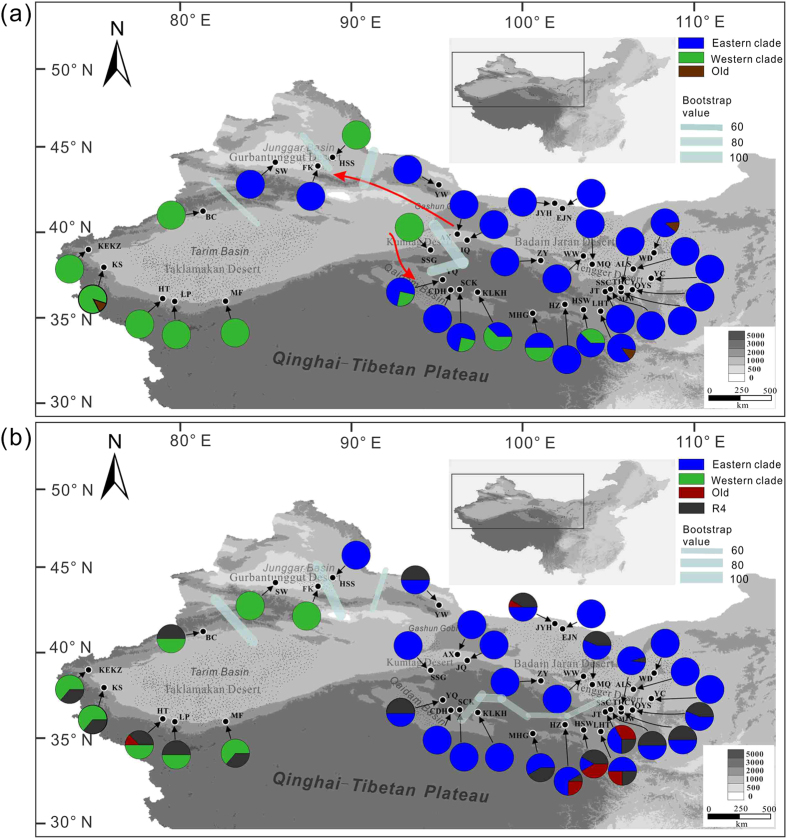
Geographical locations and haplotype distributions of the 34 *R. soongarica* populations examined in this study. (**a**) cpDNA chlorotype distribution; (**b**) ITS ribotype distribution. Population locations are presented as small black spots with white margins; slice sizes are proportional to the frequency of the haplotypes (eastern or western clade). The colors of the pie diagrams correspond to the clades of the network genealogies in Fig. 1. “Old” refers to C26, C39 and C24. The gene flow along the East Asian winter monsoon (EAWM) and East Asian summer monsoon (EASM) directions are displayed with red arrow lines in (**a**). In addition, genetic barriers are superimposed on the map between populations using a gray bold line, with the width of the barrier lines representing the bootstrap values. These figures were drawn by H.X.Y. using CorelDraw X6 (Corel Corporation, Ottawa, Canada) after being originated from the software packages Diva-GIS version 7.5.0 (http://www.diva-gis.org/) loading with the Chinese map.

Five cpDNA fragments and one nrITS fragments for a total of 272 in-group and 3 out-group individuals were sequenced. The sequences obtained from five cpDNA fragments were concatenated into a total length of 3,836 bps, with 41 chlorotypes defined by 44 polymorphic sites, including two indels (Supplementary Table S1). A total of 65 ribotypes (59 ribotypes from in-groups and 6 ribotypes from out-groups) were detected from the nrITS fragment (662 bps). Accordingly, the genealogical topology of chlorotypes and ribotypes were both clustered into two major lineages from the out-groups, and this clustering was strongly supported by the maximum likelihood (ML) trees (Fig. 1; Supplementary Fig. S1a). All chlorotypes and ribotypes from the two lineages were then projected to the population location on the geographical map (Fig. 2a,b). Most of the western populations harbored the chlorotypes and ribotypes from the western lineages, whereas most of the eastern populations were dominated by the chlorotypes and ribotypes from the eastern lineages.

Chlorotypes C1 and C9, which coalesced to chlorotype C39 connecting with the out-group, could be the most recent common ancestral chlorotypes for the eastern and western clades, respectively (Fig. 1a; Supplementary Fig. S1a). Chlorotype C1 was found in most of the populations from the eastern group BJ-TD (grouping refers to Materials and Methods), whereas C9 was dominant in the western group TaD. Interestingly, chlorotypes C1 and C9 were both found in the populations from the QB and KD-GG groups. Four of the five populations from the group QB and one marginal population HSW from the group BJ-TD near the QB harbored both the western and eastern chlorotypes (Fig. 2a; Supplementary Table S2), which suggested that the maternal genetic admixture occurred along the populations of the QB “corridor”. In the KD-GG region, however, one population SSG was fixed with the western chlorotype C9, while the other three populations were fixed with the eastern chlorotypes (Fig. 2a; Supplementary Table S2), indicating that strong genetic structures were characteristics of chloroplast DNA in the group KD-GG.

Similar to the chlorotypes, most of the ribotypes could be divided into two lineages, except for the ancestral ribotype R4 being shared among all populations from the eastern and western regions (Fig. 1b), which showed incomplete divergence and/or imcomplete lineage sorting on nrITS within this species. However, we failed to detect the genetic admixture occurred on the nuclear genome in the group QB, as none of western ribotypes but ancestral ribotype R4 and the eastern ribotypes were found in all the populations from the group QB (Fig. 2b). Furthermore, we could not detect genetic structure on the nuclear genome within the group KD-GG, as the population SSG and the other three populations from the KD-GG region were all fixed with the eastern ribotypes and R4.

As noted above, all chlorotypes and ribotypes found in the group GuD were derived from both lineages (Fig. 1a,b). Interestingly, in this region, two populations (SW and FK) were dominated by the western chlorotypes and eastern ribotypes, whereas the other population, HSS, was dominated by the eastern chlorotypes and western ribotypes (Fig. 2a,b). These results indicated that *R. soongarica* recently colonized into the GuD region from two directions.

### Divergence and diversification of chlorotype lineages.

**Figure 3:**
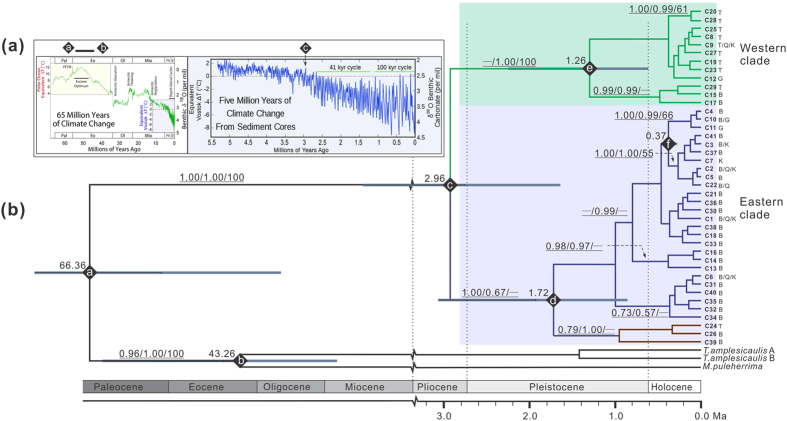
BEAST-generated maximum clade credibility tree of 41 chlorotypes (C1-C41) identified in *R. soongarica* and 3 chlorotypes identified in out-groups using the concatenated dataset with five cpDNA fragments. (**a**) PETM events (Zachos *et al.*, 2001); (**b**) BEAST tree with bootstrap support values (%) from 1,000 replicates (maximum likelihood analysis using PhyML software/posterior probabilities (Bayesian inference)/posterior probabilities (maximum likelihood using PAUP analysis) added above each branch with higher support. The node age (Mya) estimates are marked with letters of the alphabet; (**a**–**f**) represent the coalescence time of each major clade, and the length of the blue bars represents 95% highest posterior density. The capital letters at the end of each branch representing the original groups, e.g. Taklamakan Desert (T), Gurbantunggut Desert (G), Qaidam Basin (Q), Kumtag Desert and Gashun Gobi (K), and Badain Jaran and Tengger Desert (B).

Due to insufficiency of pollen or fossil records of *Reaumuria*, we carefully chose an average mutation rate of 4.87 × 10^–10^ substitutions per site per year (s/s/y) for chloroplast genome, which is suitable for desert shrub taxa^27^, to estimate the divergence time between the genealogical lineages. According to the BEAST analyses of cpDNA based on the above mutation rate, the intraspecific divergence time between the eastern and western lineages can be dated to ca. 2.96 Mya (95% HPD, 1.45–5.50 Mya, point “c” in Fig. 3), corresponding to the last peak of higher temperature during the Pliocene^28^ and/or the prominent uplift of the QTP^10^. The eastern lineage coalesced at ca. 1.72 Mya (95% HPD, 0.83–3.13 Mya point “d” in Fig. 3), considerably earlier than the western lineage at ca. 1.26 Mya (95% HPD, 0.52–2.46 Mya point “e” in Fig. 3), suggesting the asynchronous lineage diversifications of *R. soongarica* between the two regions (Badain Jaran and Tengger deserts and Taklamakan desert) before the Middle Pleistocene. Interestingly, in the group GuD, the presence of the west-like chlorotype C12 and the east-like C10 and C11 could be dated to later than ca. 0.37 Mya (point “f” in Fig. 3), indicating that the bi-directional colonization of *R. soongarica* could have occurred in the Gurbantunggut desert during the Late Pleistocene.

### Genetic isolation and gene flow

The overall genetic diversity (*H*_T_ = 0.934) of *R. soongarica* on cpDNA was considerably higher than the average diversity within populations (*H*_S_ = 0.527), which was concordant with the significant phylogeographical structure found on cpDNA (*N*_ST_ = 0.612 with *P* = 0.04, *G*_ST_ = 0.436 with *P* = 0.04, Table 1) and on nrITS (*N*_ST_ = 0.386 with *P* = 0.03, *G*_ST_ = 0.279 with *P* = 0.02; Supplementary Table S3). A hierarchical AMOVA indicated that the molecular variation among five groups (48.04%) was nearly two-fold higher than that within populations (24.74%, Table 1). In addition, strong significant pairwise genetic differentiation (Φ_ST_) was found between the group TaD and the other four groups (from 0.675 to 0.819, Table 2), which supported the geographical isolation of group TaD. This finding suggested that the gene flow from other groups couldn’t have affected the population diversity within group TaD. In contrast, negligible pairwise Φ_ST_ values were found between the three latitudinal groups (Φ_ST (QB, KD-GG)_ = 0.005, Φ_ST (QB, GuD)_ = 0.084, Φ_ST (GuD, KD-GG)_ = 0.108; Table 2). This finding supported the hypothesis that frequent latitudinal gene flow on chloroplast genome occurred among these three groups during the evolutionary history of *R. soongarica*.

Significant population structure was also found within the groups GuD and KD-GG (*H*s = 0.083 and 0.179, *F*_ST_ = 0.993 and 0.961, respectively; Table 1). In contrast, the highest genetic diversity within populations (*H*s = 0.629) and the lowest genetic differentiation (*F*_ST_ = 0.191) were found within the group QB, supporting the frequent maternal gene flow among the populations within this group (Fig. 2a; Table 1).

To determine whether genetic differentiation was significantly related to physical distance (isolation by distance), Mantel tests were conducted along the latitudinal and longitudinal directions both on maternally inherited cpDNA and bi-parentally inherited nrITS. Significant genetic isolation by physical distance was found along both latitudinal and longitudinal directions on nrITS (r = 0.430, *P* < 0.001; r = 0.794, *p* < 0.001, respectively), and along the longitudinal direction on cpDNA (r = 0.609, *p* < 0.001; Table 3). However, no significant genetic isolation was detected along the latitudinal direction on cpDNA (r = 0.262, *P* = 0.110; Table 3). This finding implied that the EAWM played a crucial role in enhancing the maternal gene flow, which minimized the genetic differentiation between populations along the latitudinal direction.

### Regional population dynamics

**Figure 4:**
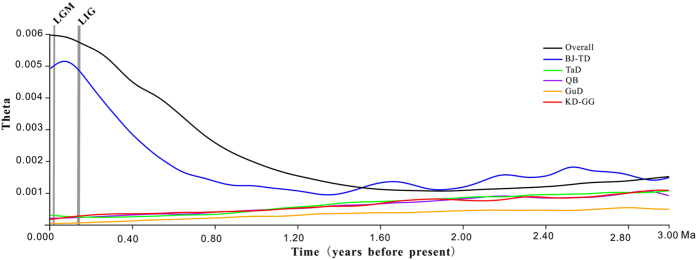
Skyline plots with MIGRATE-N estimated for each group of *R. soongarica*: x-axis, time, years before present (Mya); y-axis, theta (θ = 2*N*e*u*). The curves shown in various colors are plotted for the total populations and separated populations distributed in BJ-TD, GuD, TaD, QB and KD-GG regions.

A marginally significant negative value of Tajima’s *D* (–1.27, *P* = 0.070, Table 1) and MDA results (Supplementary Fig. S2) implied that the overall population of *R. soongarica* underwent a complex expansion of its population structure. Significant negative values of Tajima’s *D* (−1.55, *P* = 0.035) and Fu’s *F*s (−12.13, *P* < 0.001) strongly confirmed that the eastern group BJ-TD underwent a population expansion, whereas the western group TaD remained comparatively stable, as only Tajima’s *D* was significant negative (−1.72, *P* = 0.021; Table 1). The skyline plots with MIGRATE-N analysis showed that the population size of the overall *R. soongarica* populations had been increasing significantly (Fig. 4; Supplementary Fig. S3a). In the separate groups, the population size of group BJ-TD increasing more rapidly and reached a maximum at 80 Ka, whereas group TaD exhibited a slower population growth relatively recently (Fig. 4; Supplementary Fig. S4b,d). However, population decreasing was found in the three other groups with genetic admixture/structure: GuD, QB and KD-GG (Fig. 4; Supplementary Fig. S4c,e,f).

### Distribution range shifts in the past

**Figure 5:**
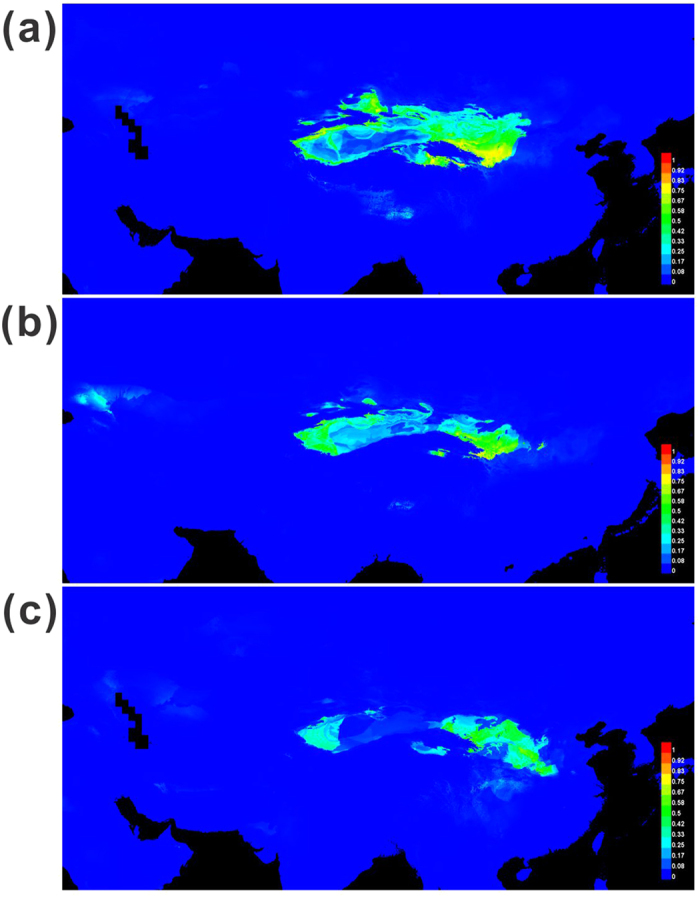
Predicted distributions of *R. soongarica* based on ecological niche modeling using Maxent. Predicted distributions are shown for (**a**) the present time, (**b**) the LGM period, and (**c**) the LIG period.

The ENM simulations based on Maxent supported the present distribution range of *R. soongarica*, which covers nearly all the desert regions across ACA (Fig. 5a). Our modeling data indicated that Bio19 and Bio11, namely, the precipitation and average temperature during the coldest season, together represented more than 60% of the niche contribution and over 80% of the permutation importance (Supplementary Table S4). These results indicated that the EAWM markedly influenced the distribution range of *R. soongarica*. Compared with the present distribution, the area of suitable habitat determining the range of *R. soongarica* was extremely contracted and fragmented by the QTP during LGM period, and this tendency further strengthened during LIG period (Fig. 5b,c). However, the local distribution range fluctuated asynchronously: the western region experienced range expansion, and the eastern region underwent more frequent range shifts, following LIG period (Fig. 5b,c). These results indicated that the paleoclimate change could have further driven the habitat fragmentation of *R. soongarica* and that the EAMS could have markedly affected vegetation distribution in different ways in the two local regions.

## Discussion

Based on thorough investigation on genetic structure and distribution range shifts under monsoonal climate scenarios, we intend to understand the mechanism of population divergence and population dynamics of *R. soongarica*, a Tertiary relic constructive zonal shrub species widely distributed across all the desert ecosystems in ACA. As the formation and evolution of ACA had been affected by the development of EAMS since Pliocene^7^, the present work supported an idea model to discuss the role of monsoonal climatic changes in shaping the habitat fragmentation which triggered the intraspecific divergence/speciation of the dominant species of ACA.

The cpDNA genealogy network and phyML tree (Fig. 1a; Supplementary Fig. S1a) suggested that *R. soongarica* diverged into two contrasting lineages. This finding is consistent with our previous study^21^. The chlorotypes from the western lineage were mainly distributed in the group TaD, whereas those from the eastern lineage were dominant in the group BJ-TD (Fig. 2a). Marked genetic differentiation between these two groups was found in both nrITS and cpDNA (Table 2). Due to the effective population size of the biparentally inherited nuclear genome is twofold larger than that of the uniparentally inherited chloroplast genome, we found incomplete divergence on nuclear ribosome DNA. The most recent common ancestral ribotype, R4, was shared among all groups, excluding GuD (Fig. 1b). However, we detected significant genetic barriers on nrITS between the group TaD and the other groups (Fig. 2b). This marked genetic differentiation between the eastern and western populations in ACA is not specific to *R. soongarica.* It has also been found in some sympatric plant species such as *Nitraria sphaerocarpa*^18^ and *N. tangutorum* (unpublished data). These findings suggest that geological events common to all populations could have impacted the genetic differentiation of the plants endemic to ACA.

**Figure 6:**
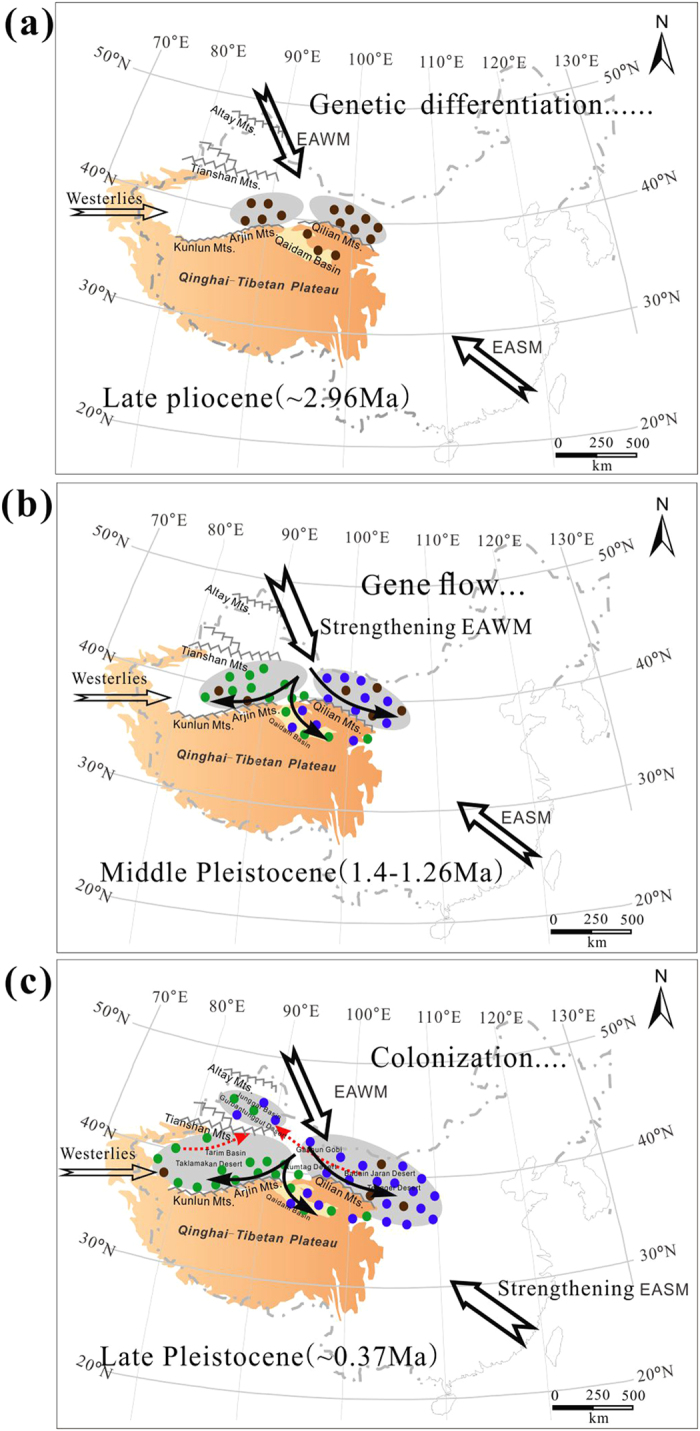
Historical schemes of spatial-temporal evolution historical schemes of paleogeologies and paleoclimatics and the corresponding genetic response of *R. soongarica* in the ACA region. (**a**) ca. 2.96 Mya: genetic differentiation occurred within *R. soongarica* triggered by the uplift of the northeastern QTP and EAWM formation; (**b**) 1.4 ~ 1.26 Mya: maternal gene flow from the KD-GG region (SSG) to the QB and TaD regions under the driving force of the strengthening EAWM; the red line represents the orientation of gene flow; and (**c**) ca. 0.37 Mya: bi-directional colonization and aridification of the GuD region occurred; the dashed red line represents the directions of colonization. Color coding was consistent with Fig. 2 as follows: the brown spot represents the ancestor of *R. soongarica*, the blue pie represents the haplotyps from the eastern clade, and the green pie represents ones from the western clade. The shadow represents the expanding arid regions responding to the habitat changes of *R. soongarica* during different geological periods. These figures were drawn by H.X.Y. using CorelDraw X6 (Corel Corporation, Ottawa, Canada).

To further clarify the cause of the intraspecific divergence between the two groups, molecular dating was conducted with a relaxed clock method via Bayesian simulation based on substitution rate of 4.87 × 10^−10^ s/s/y for the chloroplast genome^27^. The divergence between the two out-group genera could be dated to ca. 43 Mya (95% HPD: 16.63–74.68 Mya, point “b” in Fig. 3b). This result did not conflict with the earliest fossil record of the genus *Tamarix*^29^. The crown age of genus *Reaumuria* and the out-groups could be dated to ca. 66 Mya (95% HPD: 111–27 Mya, point “a” in Fig. 3b). It is consistent with a previous report^30^, and corresponding to the period of Paleocene-Eocene Thermal Maximum (PETM, Fig. 3a), when a sharp increase in concentration of atmospheric CO_2_ caused the global surface temperature to increase by approximately 5–6 °C^28^. The intraspecific divergence of *R. soongarica* between the eastern and western lineages could be dated to ca. 2.96 Mya (95% HPD: 1.45–5.5 Mya, point “c” in Fig. 3b). This time point is close to the strong tectonic uplift of QTP, especially, a rapid uplift of Qaidam Basin (the northeastern QTP) during the late Pliocene^7^,^14^. Such a rapid uplift is consistent with our molecular dating of the intraspecific divergence of *R. soongarica* to a period prior to the beginning of the dramatic glacial and interglacial oscillations of the global climate (Fig. 6a).

However, the abrupt uplift of the QTP had also caused the intensification of EAMS at ca. 3.6 to 2.6 Mya. Actually, based on ENM simulation of the potential distribution range of *R. soongarica* during the LGM (EAWM dominated) and LIG (EASM dominated), both the glacial and interglacial climates could have greatly contracted the habitat of this species and finally fragmented its distribution range (Fig. 5b,c). On the other hand, the present distribution of *R. soongarica* was not fragmented at all (Fig. 5a), indicating that the two lineages of *R. soongarica* should have contacted easily and the two reciprocal habitats could have merged together under the moderate climatic conditions. Therefore, we could not arbitrarily conclude that the geological event was the only condition that triggered the intraspecific divergence of *R. soongarica*. Based on the considerations above, we proposed that once the isolation occurred as a result of geological cause, the cycles of the monsoonal climatic oscillations was most likely to play a key role in the habitat fragmentation and intraspecific divergence of *R. soongarica*. After *R. soongarica* diverged into two regional lineages, further diversification of two lineages began ca. 1.72 Mya (Eastern clade, point “d” in Fig. 3b) and 1.26 Mya (Western clade, point “e” in Fig. 3b), which are fairly concordant with the two main turning points of the EAMS^31^,^32^. Stratigraphic data from loess deposits and NW-Pacific ocean deposits suggested that the EAWM steadily strengthened during Pleistocene, whereas the EASM underwent stepwise weakening until ca. 1.8 Mya, when the EASM was increasingly restrengthened. Considering the more fragmented habitat of *R. soongarica* in the interglacial period such as LIG (Fig. 5c), we speculated that the strengthening EASM of the warming stage ca.1.8 Mya could have fragmented the habitat of *R. soongarica* and accelerated the diversification of the eastern lineage. However, the western lineage could not start diversification until ca. 1.3 Mya, when the restrengthened EAWM effectively overwhelmed the EASM and induced the further aridification of ACA^32^. Therefore, we suspected that the EAWM played a crucial role in the diversification of the western lineage of *R. soongarica*.

Since the asynchronous diversification happened between the two lineages of *R. soongarica*, could the two regional groups have undergone the similar population dynamics? In fact, from the skyline plots obtained with MIGRATE-N, we found a significant increase of the population size in the eastern groups of *R. soongarica* (Fig. 4), concordant to significant negative values of the Tajima’s *D* and Fu’s *F*s and star-like shape of the genealogy network (Fig. 1; Table 1). Comparatively, the population size of the western group TaD was much more stable (Fig. 4; Supplementary Fig. S4d). Considering the distribution range of *R. soongarica* expanding slightly in the western region from LIG to the present based on ENM analysis (Fig. 5b,c), the stable population dynamic of the western group TaD could be related to the geographical isolation caused by the Tianshan-Kunlun-Arjin Mountains and equable climate during the late Pleistocene^15^,^33^.

Given that the population size of the western group remained stable while its distribution range expanded, and that the eastern group underwent a remarkable population growth while its distribution range shifted significantly (Figs 4 and 5b,c), the population expansion of the eastern group could be attributed to the frequent range shifts rather than to habitat range expansion. The frequent range shifts during the Pleistocene exerted strong selective forces that facilitated the accumulation of new mutants in the local populations, and consequently induced the effective population to grow more rapidly in the eastern climate-sensitive group than in the western climate-stable group. Previous studies have suggested that during the Pleistocene climate oscillation, population growth was quite common for large-sized species, such as conifers endemic to the climatic-sensitive region, e.g., the southeast QTP^4^,^34^ and other plants^16^ and animals^35^ endemic to ACA and adjacent regions. Unfortunately, these studies failed to interpret the potential effects of local climate change on the speed and scale of population growth. Here, based on the comparison between the regional groups of the population dynamics and range shifts of *R. soongarica*, we presented a novel example that the populations in the monsoonal climate zone tended to grow more rapidly than those far from it.

Additionally, an interesting geographical pattern of chlorotypes of *R. soongarica* showed that frequent genetic admixture of the two lineages occurred in most populations within the QB “corridor” (Fig. 2a). On the other hand, we detected negligible pairwise genetic differentiation (Φ_ST_) among the groups GuD, Ku-GG and QB, which suggested that the latitudinal gene flow had been markedly enhanced (Table 2). To further verify the effects of the EAMS on gene flow among the groups of *R. soongarica*, we conducted Mantel tests on maternally inherited cpDNA and bi-parentally inherited nrITS along different monsoon directions. Our data strongly supported that the EAWM decisively accelerated maternal gene flow along the latitudinal direction (Table 3). Due to the hairy and light characteristics for the mature seeds of *R. soongarica*, it is reasonable that the seeds of *R. soongarica* could be easily dispersed by the EAWM during autumn, accelerating maternal gene flow across the QB “corridor.” Gene flow can minimize intra- and inter-specific genetic differentiation and induce the accumulation of heterozygosity, facilitating the colonization of new habitats and promoting adaptation to climate change^36^,^37^. Many well-adapted plant species such as forest trees would utilize wind systems for seeds or pollen dispersal to increase gene flow between diverged populations^38^,^39^,^40^. By dating the historical maternal gene flow occurred among the groups of *R. soongarica*, we concluded that the EAWM intensified at least after the origin of the chlorotype C9 in the western group (ca. 1.4 Mya, more reasonable after 1.3 Mya, Figs 1a and 6b). In fact, many stratigraphic investigations across the Loess Plateau have recorded that the EAWM intensified after 1.3 Mya^7^,^8^,^32^. Up to date, empirical data are lacking except our new evidence from the desert plant *R. soongarica* on the scale and direction of the gene flow induced by the EAWM.

More interestingly, phylogenetic data of chlorotypes suggested that *R. soongarica* has recently (since ca. 0.37 Mya, point “f” in Fig. 3b) colonized into the GuD region, which was concordant to that the Gurbantunggut Desert expanded due to the intensification of the EAWM ca. 0.4 Mya^41^. This phylogeographic pattern indicates that the aridification of ACA during this time had expanded to the Gurbantunggut Desert region, which had supplied new habitats for the xerophyte *R. soongarica* (Fig. 6c). The stepwise colonization of plant species has also been found in an alpine conifer species endemic to the QTP^42^,^43^. In this study, we provided the first evidence for the stepwise aridification processes of ACA from a molecular phylogeographic perspective and confirmed that the formation time of Gurbantunggut Desert was later than the other deserts, e.g., Badain Jaran and Tengger deserts and Taklimakan Desert in ACA^44^ The onset of the desertification of ACA has been debated for several decades, with the time estimates ranging from ca. 5.3 Mya to 0.85 Mya^45^,^46^,^47^,^48^,^49^. The regional asynchronous diversification of the constructive shrub *R. soongarica* indicated that the desertification of the eastern Badain Jaran and Tengger deserts regions could have occurred at 1.72 Mya, the western Taklimakan Desert could have formed ca. 1.26 Mya, and the northern Gurbantunggut Desert could have formed ca. 0.4 Mya. Of course, as the corresponding 95% CIs of dating were still wide, more evidence from fossil and molecular records are needed to clarify the accurate formation time of the deserts across ACA.

In summary, our study supported a unique case focused on evaluating the roles of EAMS in impacting the evolutionary history of constructive shrub species endemic to ACA, by triggering the intraspecific divergence between the longitudinal populations and enhancing gene flow among the latitudinal populations. Furthermore, this work supplied new molecular evidence for understanding the spatial and temporal evolution of ACA from a phylogeographic view point.

## Methods

### Population sampling

A total of 272 individuals were sampled from 34 populations across the ACA geographical ranges (Supplementary Fig. S5; Table S5). Six of these populations were collected in Taklimakan Desert (TaD) region, three in Gurbantunggut Desert (GuD) region, five in Qaidam Basin (QB) region, four in Kumtag Desert and Gashun Gobi (KD-GG) region, and 16 in Hexi corridor and Badain Jaran - Tengger deserts (BJ-TD) regions. Additionally, three individuals of *Tamarix amplexicaulis* and *Myricaria pulcherrima* were collected as outgroups to *Reaumuria*^24^. In each population, samples of fresh leaves were collected from eight individuals at least 30 meters apart to reduce sampling bias and dried by silica gel. The information on the location of each population, including latitude, longitude and altitude, were recorded with an e-Trex Global Positioning System (Garmin, Taiwan; Supplementary Table S5). All of the sampled voucher specimens were deposited in the Key Laboratory of Stress Physiology and Ecology in Cold and Arid Regions, CAREERI, CAS.

### DNA extraction, PCR amplification and sequencing

The total genomic DNA was extracted using DNeasy Plant Kits (Qiagen, Valencia, CA, USA) and then stored at −30 °C in a refrigerator. Six individuals from populations separated by long distances were sequenced to verify the polymorphic sites from 23 pairs of cpDNA fragments (Supplementary Table S6). Due to the maximum polymorphic sites, five cpDNA fragments (*pet*B*-pet*D*, trn*S2*-trn*G2*, atp*H*-atp*I*, ndh*A and *pet*N*-psb*M) and one nrITS fragment were selected for full phylogeographic surveys (Supplementary Table S7), and all six fragments were amplified and sequenced in the 272 individuals of *R. soongarica*. DNA amplifications were performed in a Gene-Amp PCR system 9700 (Applied Biosystems, USA) with the thermal program referred to Supplementary Method S1. After purification by TIAN quick Midi Purification Kits reagent (TIANGEN, Beijing, China), sequencing with forward and reverse primers in all individuals was performed using an ABI 3130xl Genetic Analyzer (Applied Biosystems, USA) with an ABI Prism BigDye Terminator Cycle version 3.1 Sequencing Kit according to the manufacturer’s instructions. All sequences were double checked by eye in BioEdit version 7.0.5.3 and initially aligned by ClustalX version 1.81^50^. All newly obtained sequences for each variety have been deposited in GenBank (accessions: KM242133-KM242261).

### Nucleotide diversity and genealogy

Sequences from each cpDNA fragment were concatenated into a supergene matrix by the program DnaSP Version 5.0^51^. For nrITS sequences, if double peaks occurred in the same position in both directions and the weakest signal reached at least 25% of the strongest signal, we considered the site to be heterozygous^52^. Each two ribotypes of all nrITS in heterozygous individuals were separated under the default parameters with PHASE implemented in the program DnaSP Version 5.0. The molecular diversity for the concatenated sequences of five cpDNA fragments with the numbers of haplotypes (*n*), segregating sites (*s*), total haplotype diversity (*h*_d_) and nucleotide diversity (*p*_i_) for each population were also estimated with DnaSP Version 5.0. The genealogical topologies of chlorotypes and ribotypes were constructed using the program NETWORK Version 4.2.0.1 with a median-joining model (available at http://www.fluxus-engineering.com/)^53^.

### Population genetic structure

Population gene diversity (*H*_S_, *H*_T_) and between-population divergence (*G*_ST_, *N*_ST_) were estimated for each region based on the cpDNA using the program PERMUT with 1,000 permutation tests (available at http://www.pierroton.inra. fr/genetics/labo/Software/PermutCpSSR)^54^. In addition, spatial genetic structures of chlorotypes and ribotypes were analyzed with SAMOVA Version 1.0^55^, using a simulated annealing procedure to identify the number of population groups (*K*) that were geographically homogeneous and maximally differentiated from each other. According to the various geographical locations and SAMOVA results, all populations of *R. soongarica* were divided into five groups as follows: QB, BJ-TD, TaD, GuD and KD-GG. Notably, the populations from the KD-GG group were considered one group due to the geological “junction” location of the GuD, TaD, QB and BJ-TD groups. The populations collected from the Hexi Corridor and from the Badain Jaran-Tengger deserts were combined as the BJ-TD group according to the analysis of the initial polymorphism survey and geographical locations (Supplementary Fig. S5). Further genetic differentiation among groups (*F*_CT_), among populations within groups (*F*_SC_), and within populations (*F*_ST_) was calculated with a hierarchical analyses of molecular variance (AMOVA) using Arlequin version 3.11^56^, with significance tests of variance components and differentiation indices with 1,000 permutations. Pairwise differentiations (Φ_ST_) between all pairs of groups were also estimated with 1,000 permutations.

We used the software BARRIER version 2.2^57^ to identify the areas of genetic discontinuity or barriers to gene flow across the sampled species’ ranges. One hundred bootstrapped datasets were generated by re-sampling individuals within populations with replacement using PopTools version 3.2.5 (available at http://www.poptools.org/). Under ideal circumstance, the pairwise genetic differentiation between the populations could not reject the Mantel test supporting isolation by distance^58^. To test the significance of the impact of wind systems on maternal gene flow limited by physical distance, Mantel tests along different East Asian monsoon directions were performed in IBDWS (available at http://ibdws.sdsu.edu/~ibdws/) based on the pairwise *F*_ST_ between populations using 1,000 randomizations^59^.

### Phylogenetic analyses and estimates of divergence time

The phylogenetic relationships among chlorotypes and ribotypes were reconstructed by PhyML version 3.0 with maximum likelihood (ML)^60^ and by MrBayes Version 3.2.1 with mixed models using Bayesian inference (BI) and MCMC sampling^61^,^62^, respectively. The best-fit nucleotide substitution model for the codon position was evaluated by the Akaike Information Criterion^63^ with the program jModeltest Version 0.1.1 under the ML criterion^64^ and then by GTR+G and TrN+I+G models for cpDNA and nrITS, respectively. The ML analyses were conducted with the heuristic search option over 100 random-taxon-addition replicates with tree bisection–reconnection (TBR) branch swapping. Trees were sampled for every 100 generations, resulting in 100,000 trees, and the first 25% were discarded as burn-in. Bayesian analyses were performed over 50 million generations with one cold and three incrementally heated Markov Chain Monte Carlo (MCMC) analyses, starting from a random tree and sampling every 1,000 generations. The first 10% of trees were treated as burn-in and discarded, and all remaining trees were used to construct a Bayesian consensus tree. All trees were then modified using FigTree version 1.3.1^65^.

To estimate the divergence time between the genealogical lineages, we carefully chose a mutation rate for cpDNA of 4.87 × 10^−10^ s/s/y, which is suitable for shrub species^27^. Since neither any pollen nor fossil records of Reaumuria could be found from the deposit strata published, we chose two closely related genera (*Tamarix* and *Myricaria*) within Tamaraceae to calibrate the molecular clock. Based on this mutation rate and its assumed standard deviation (10%), the divergence time and coalescence time of lineages were estimated using a Bayesian approach implemented in BEAST Version 1.7.5 with the criteria set by BEAUti (available at http://BEAST.bio.ed.ac.uk)^65^. All simulations were based on an uncorrected log-normal relaxed clock distribution of branch lengths, started with a prior constant population size and a randomly generated starting tree. The posterior distributions of the parameters in the MCMC analyses were approximated with 80 million steps in each analysis and sampled every 8,000 generations. Convergence of the parameters sampled was checked with the program TRACER version 1.5 to examine the highest effective sampling size values (ESSs > 200) for all parameters^66^, and the burn-in steps were discarded to obtain an estimation of the posterior probability distribution of divergence time at the relevant node. The maximum lineage credibility tree was then summarized by TreeAnnotator version 1.7.5 (available at http://BEAST.bio.ed.ac.uk/TreeAnnotator/).

### Inference of demographic history

Possible population expansions were assessed by neutrality tests with Tajima’s *D* and Fu’s *F*s and mismatch distribution analysis (Supplementary Method S2). MIGRATE-N version 3.6.4^25^ was used to estimate the variation in the historical effective population size. The default settings were conducted in the MIGRATE-N program with some modified options as follows: the Bayesian inference module; one single long run using heating with temperatures of 1.0, 1.5, 3.0 and 10,000, totaling 50,100,000 visited parameter and genealogy changes in the cold (1.0) chain; sampling 10 replicates of each 50,000 in intervals of 100; after discarding the first 100,000 visits. The mutation-scaled population size theta (θ) was displayed by the uniform prior distributions with ranges from 0 to 0.1, namely the effective population size Ne times the mutation rate μ per site and generation. Moreover, Bayesian skyline plots (BSPs) for population dynamics were also conducted on cpDNA (Supplementary Method S3).

### Ecological niche modeling

To predict the distribution of *R. soongarica* based on habitat suitability, ecological niche modeling was conducted with Maxent^26^ with the WorldClim database from 1955 to 2000 (http://www.worldclim.org/). The pairwise correlation for all 19 biological factors were tested, and 12 independent biological factors were used for further predictions. The distribution sites consisted of those examined in the field investigation together with the specimen records from the Chinese Virtual Herbarium and with coordination from the Global Positioning System (http://www.cvh.org.cn/); 75% of the sites were randomly selected to design a prediction scheme on consideration of the commission error, and the remaining 25% of the sites were used for evaluations of the goodness of the prediction.

The contemporary species-climate relationships were then projected to past climate layers to predict the potential range of the species during LGM (ca. 21,000 years BP) and LIG (ca. 120,000 – 140,000 years BP) periods. The project bioclimatic layers for LIG (resolution of 30 arc-second) and LGM (resolution of 2.5 arc-minute) were obtained from the WorldClim database version 1.4^67^. We employed a “maximum training sensitivity plus specificity” threshold to determine suitable/unsuitable habitat because sensitivity–specificity combined approaches have been suggested as good thresholds^68^. An AUC value estimation greater than 0.9 for each estimate was used as the criterion for a good model. Additionally, 100 permutations were conducted in the modeling process for LGM and LIG periods based on the MIROC model. The relative contribution of each variable was evaluated with the covariance in terms of increased predictive performance resulting from a given environmental variables. Only the ecological variables that explained greater than 10% of the prediction variance were viewed as the principal habitat suitability factors limiting the distribution range of *R. soongarica*.

## Additional Information

**How to cite this article**: Yin, H. *et al.* The role of East Asian monsoon system in shaping population divergence and dynamics of a constructive desert shrub *Reaumuria soongarica*. *Sci. Rep.* **5**, 15823; doi: 10.1038/srep15823 (2015).

## Supplementary Material

Supplementary Information

## Figures and Tables

**Table 1:** Estimates of genetic diversity and molecular variance for chlorotype sequences.

Regions	*H*_S_	*H*_T_	*G*_ST_	*N*_ST_	Source of variation	d.f.	SS	VC	PV (%)	Fixation index	*θ*_*w*_(×10^−3^)	*Ne*(×10^5^)	Neutrality tests	
													Fu’s Fs**	Tajiam’s D**
TaD	0.375 (0.086)	0.747 (0.090)	0.498 (0.171)	0.501 (0.196)	Among populations	5	14.46	0.28	29.20	*F*_ST_ =0.292*	0.7	6.67	−1.60 (0.220)	−1.72 (0.021)
					Within populations	42	28.25	0.67	70.80					
					Total	47	42.71	0.95						
GuD	0.083 (0.083)	0.708 (0.175)	0.882 (0.162)	0.985 (0.022)*	Among populations	2	90.75	5.67	99.27	*F*_ST_ =0.993*	0.6	5.75	12.41 (0.998)	1.78 (0.962)
					Within populations	21	0.88	0.04	0.73					
					Total	23	91.63	5.71						
BJ-TD	0.630 (0.062)	0.916 (0.023)*	0.313 (0.066)	0.402 (0.058)	Among *p*opulations	15	84.42	0.56	33.63	*F*_ST_ =0.336*	1.4	14.48	−12.13 (<.0001)	−1.55 (0.035)
					Within populations	112	124.75	1.11	66.37					
					Total	127	209.17	1.68						
QB	0.629 (0.045)*	0.802 (0.056)	0.216 (0.040)*	0.219 (0.009)*	Among populations	4	40.35	0.82	19.09	*F*_ST_ =0.191*	0.6	6.37	9.42 (0.993)	1.89 (0.973)
					Within populations	35	122.25	3.49	80.91					
					Total	39	162.60	4.32						
KD-GG	0.179 (0.112)	0.997 (0.047)*	0.821 (0.113)	0.923 (0.078)	Among populations	3	105.81	4.39	96.09	*F*_ST_ =0.961*	0. 6	6.67	6.72 (0.980)	1.00 (0.877)
					Within populations	28	5.00	0.18	3.91					
					Total	31	110.81	4.57						
TaD, GuD, BJ-TD, KD-GG and QB versus					Among groups	4	485.59	2.29	48.04	*F*_CT_ =0.524*				
					Among populations within groups	29	335.79	1.30	27.22	*F*_SC_ =0.753*				
					Within populations	238	281.13	1.18	24.74	*F*_ST_ =0.480*				
Total	0.527 (0.048)*	0.934 (0.011)*	0.436 (0.041)*	0.612 (0.044)*		271	1102.50	4.77			1.8	18.28	−4.68 (0.192)	−1.27 (0.070)

*H*_S_, average gene diversity within populations; *H*_T_, total gene diversity; *G*_ST_, interpopulation differentiation; *N*_ST_, number of substitution types; d.f., degrees of freedom; SS, sum of squares; VC, variance component; *F*_ST_, correlation within populations relative to total; *F*_SC_, correlation of haplotypes within groups relative to the total; *F*_CT,_ correlation within populations relative to groups; *θ*_w_, theta per site; *Ne*, estimates of the effective population size; *P*-values are enclosed in the parenthesis; *P < 0.05.

**Table 2:** Pairwise genetic differentiation (Φ_ST_) among five groups estimated from internal transcribed spacer (ITS) sequences (upper part) and cpDNA sequences (lower part) of *R. soongarica.*

	BJ-TD	KD-GG	GuD	TaD	QB
BJ-TD	—	0.064***	0.550***	0.337***	0.029*
KD-GG	0.139***	—	0.583***	0.417***	0.089***
GuD	0.322***	0.108 ^NS^	—	0.333***	0.511***
TaD	0.819***	0.675***	0.638***	—	0.311***
QB	0.212***	0.005**	0.084^NS^	0.581***	—

*P < 0.05; **P < 0.01; ***P < 0.001; NS, not significant.

**Table 3:** Mantel tests along the monsoon directions for genetic isolation by eographicl distance on cpDNA and ITS.

Directions	Population groups	cpDNA	ITS
Latitudinal (EAWM)	GuD, KD-GG and QB	**r** **= 0.262**	r **= **0.794
		***p*** **= 0.110**	*p* < 0.001
Longitudinal (Westerly)	TaD, QB, KD-GG and BJ-TD	r **= **0.609	r **= **0.430
		*p* < 0.001	*p* < 0.001
Southeastern (EASM)	GuD, KD-GG and BJ-TD	r **= **0.438	r **= **0.546
		*p* **= **0.003	*p* < 0.001

EAWM, East Asian winter monsoon; EASM, East Asian summer monsoon.
