# Supplementary material for: The role of East Asian monsoon system in shaping population divergence and dynamics of a constructive desert shrub Reaumuria soongarica
Source: Sci Rep. 2015 Oct 29;5:15823. doi: 10.1038/srep15823 (PMC4625182; doi:10.1038/srep15823)
Supplement: Supplementary Information [file srep15823-s1.doc]

Supplementary information for:

**The role of East Asian monsoon system in shaping population divergence and dynamics of a constructive desert shrub *Reaumuria soongarica***

Hengxia Yin^1, 2, §^, Xia Yan^1,3, §^, Yong Shi^1, 2^, Chaoju Qian^1, 2^, Zhonghu Li^1,4^, Wen Zhang^1,5^, Lirong Wang^1,5^, Yi Li^5^, Xiaoze Li^6^, Guoxiong Chen^1^, Xinrong Li^1, 7^, Eviatar Nevo^8^ and Xiao-Fei Ma^1*^

1 Key Laboratory of Stress Physiology and Ecology in Cold and Arid Regions, Gansu Province, Department of Ecology and Agriculture Research, Cold and Arid Regions Environmental and Engineering Research Institute, Chinese Academy of Sciences, Lanzhou 730000, Gansu, China;

2 University of Chinese Academy of Sciences, Beijing 100049, China;

3 Key Laboratory of Eco-hydrology and of Inland River Basin, Cold and Arid Regions Environmental and Engineering Research Institute, Chinese Academy of Sciences, Lanzhou 730000, Gansu, China;

4 Northwest university, Xi’an 710075, Shanxi, China;

5 College of Forestry Science, Gansu Agricultural University, Lanzhou 730000, Gansu, China;

6 Key Lab of Desert and Desertification, Cold and Arid Regions Environmental and Engineering Research Institute of Chinese Academy of Sciences, Lanzhou 730000, Gansu, China;

7 Shapotou Desert Research and Experiment Station, Cold and Arid Regions Environmental and Engineering Research Institute, Chinese Academy of Sciences, Lanzhou 730000, Gansu, China;

8 Institute of Evolution, University of Haifa, Haifa 31905, Israel.

*Corresponding. [maxiaofei@lzb.ac.cn](mailto:maxiaofei@lzb.ac.cn)

§These authors contributed equally to this work.

**
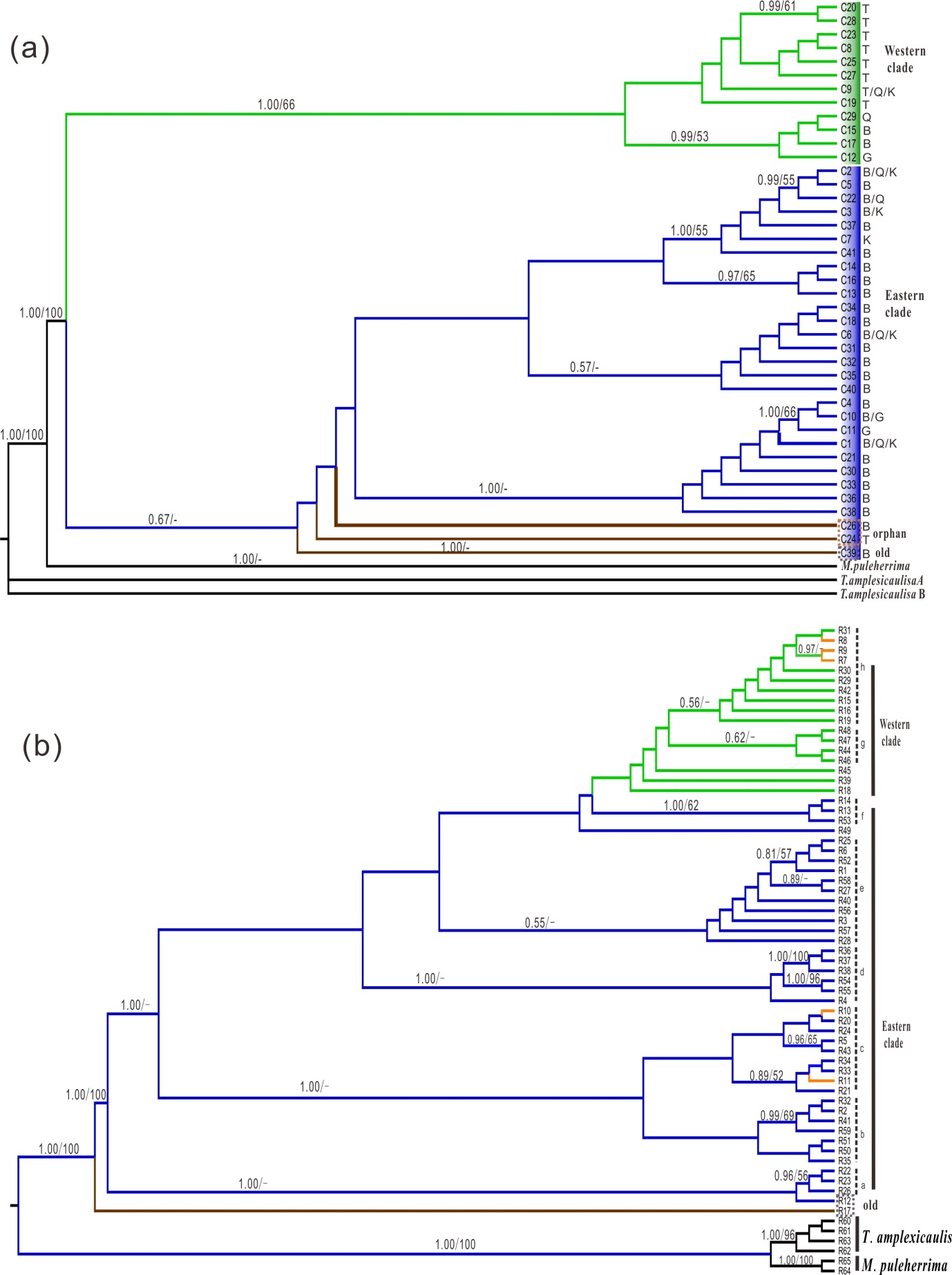
**

**Supplementary Figure S1** Phylogenetic relationships of haplotypes of *R. Soongarica*. (a) ML tree of chlorotypes; (b) ML tree of ribotypes; numbers above the tree branches indicate the bootstrap values calculated using the Bayesian inference (before slashes) and maximum likelihood methods (after slashes); Color codes define the eastern and western clades as in Fig. 1. The capital letters at the end of each branch represent the original groups, e.g. Taklamakan Desert (T), Gurbantunggut Desert (G), Qaidam Basin (Q), Kumtag Desert and Gashun Gobi (K), and Badain Jaran and Tengger Desert (B).


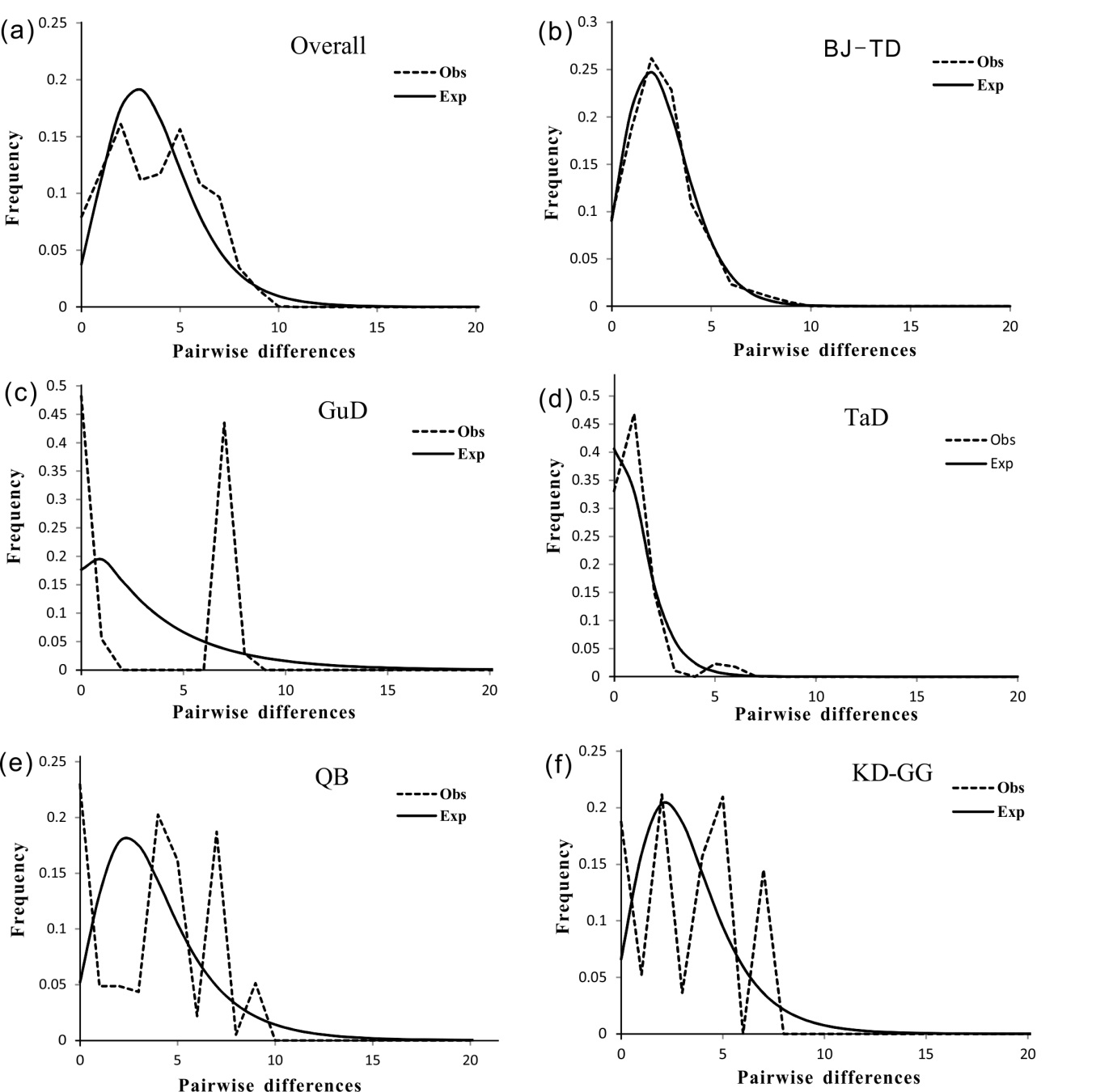


**Supplementary Figure S2** Mismatch distribution analysis(MDA) plots for cpDNA in groups: (a) *R. soongarica* as a whole, (b) populations in BJ-TD region, (c) populations in GuD region, (d) populations in TaD region, (e) populations in QB region and (f) populations in KD-GG region. The solid line represents the observed distributions of differences among chlorotypes, and the dashed line represents simulated distributions under a model of sudden (stepwise) demographic population expansion^1^.


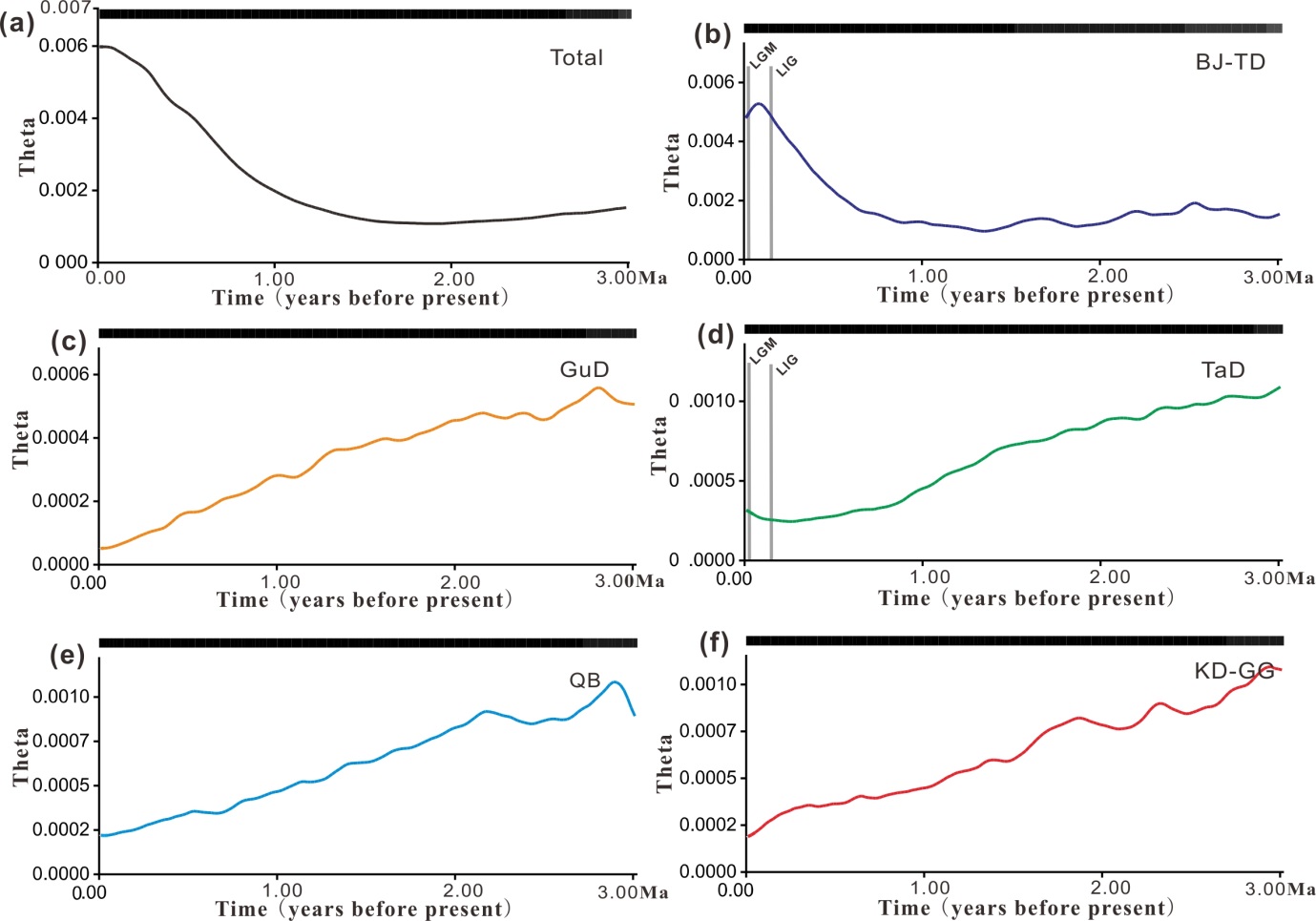


**Supplementary Figure S3** Skyline plots for population dynamics on cpDNA with MIGRATE-N in groups. x-axis: time, years before present (million years ago, Ma); y-axis: theta (Ө = 2*N*e*u*). The top of the black bar indicates the reliability; a deeper color indicates a higher confidence level. (a-f) Skyline plots for the entire population and separated populations distributed in the BJ-TD, GuD, TaD, QB, and KD-GG regions, respectively.


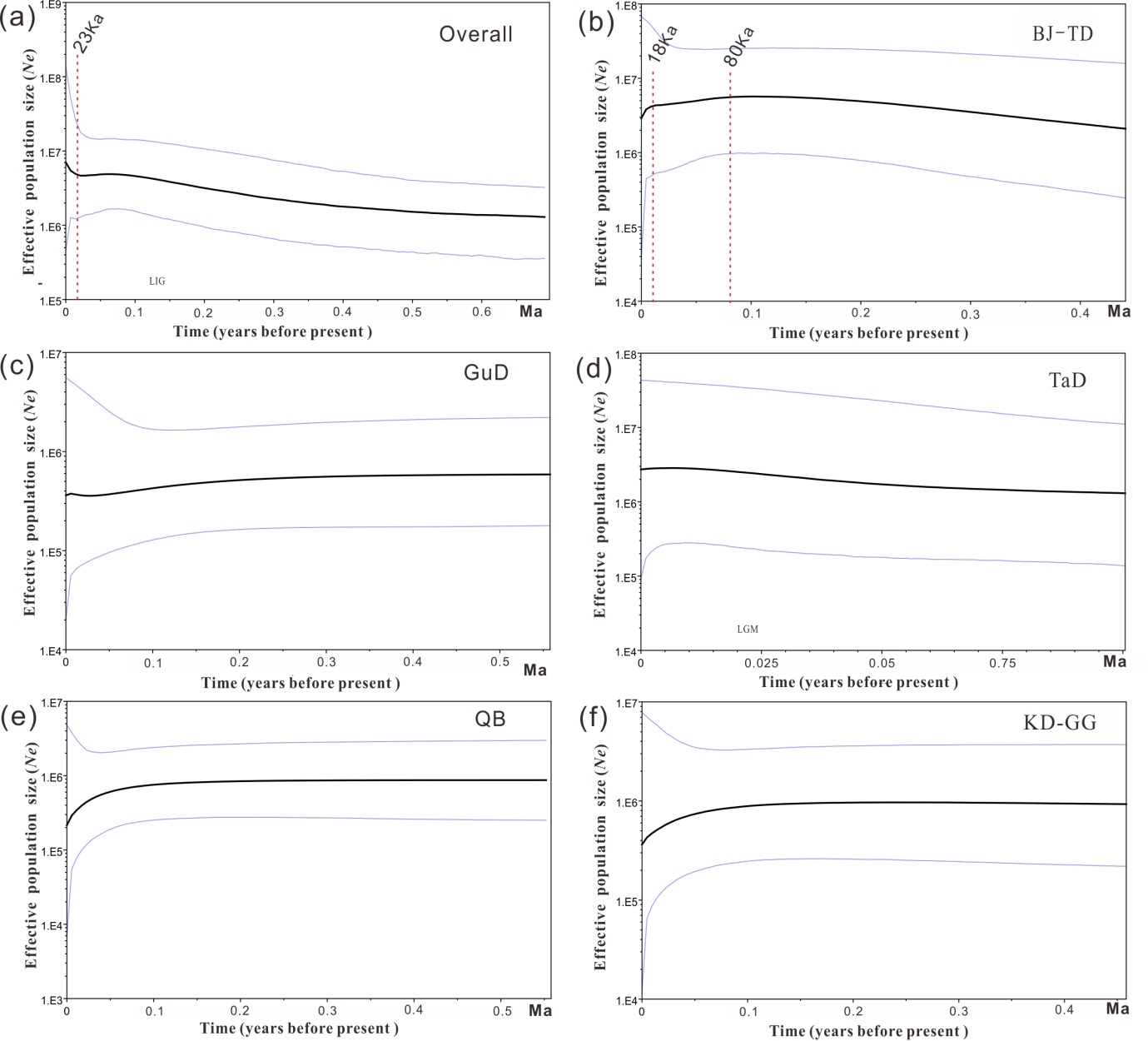


**Supplementary Figure S4** Bayesian skyline plots (BSPs) for population dynamics on cpDNA with BEAST in groups. x-axis: x-axis: time, years before present (Ma); y-axis, effective population size (*N*e*τ*, the product of effective population size and generation length). The mean estimate and both 95% HPD limits are presented. (a-f) BSP for the entire population and populations distributed in the BJ-TD, GuD, TaD, QB, and KD-GG regions, respectively.


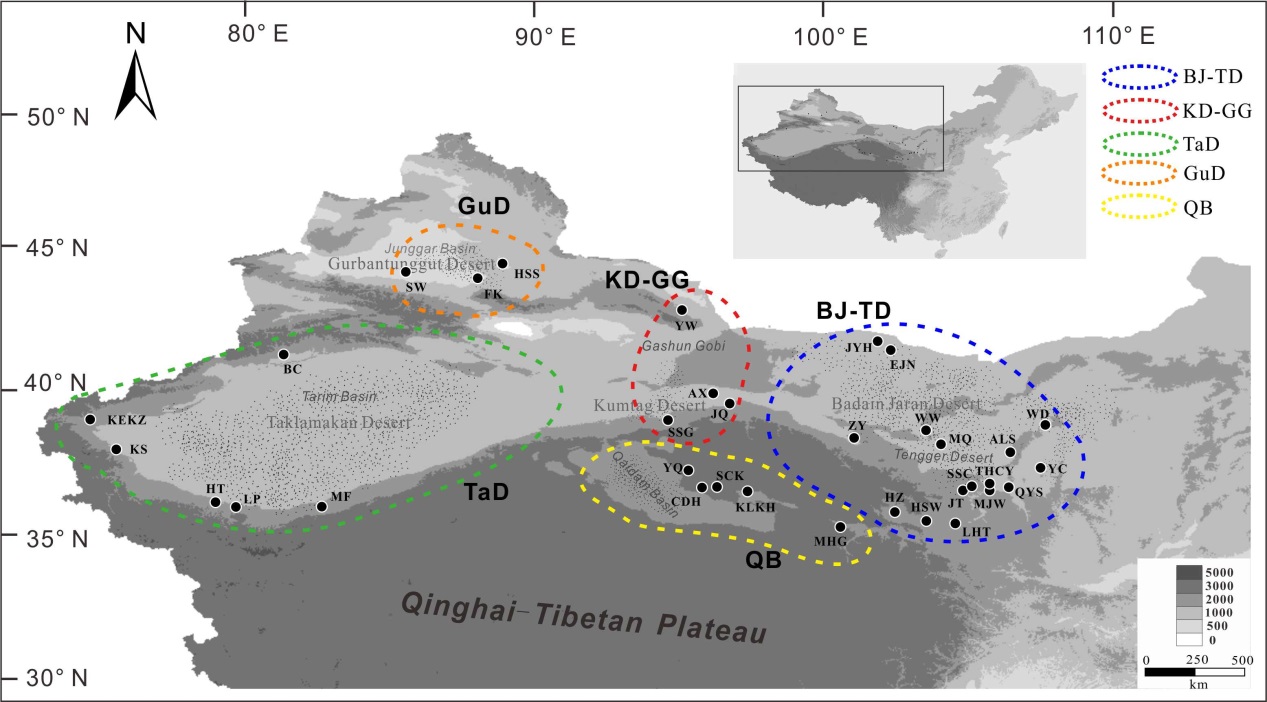


**Supplementary Figure S5** Geological location for populations and groups of *R. soongarica* sampled in this study. This figure was drawn by H.X.Y. using CorelDraw X6 (Corel Corporation, Ottawa, Canada) after being originated from the software packages Diva-GIS version 7.5.0 (<http://www.diva-gis.org/>) loading with the Chinese map.

**Supplementary Table S1** Variable sites of the aligned sequences in total of forty-one ribotypes.

| Chlorotypes | Nucleotide variable positions | | | | | | | | | | | | | | | | | | | | | | | | | | | | | | | | | | | | | | | | | | | | |
| --- | --- | --- | --- | --- | --- | --- | --- | --- | --- | --- | --- | --- | --- | --- | --- | --- | --- | --- | --- | --- | --- | --- | --- | --- | --- | --- | --- | --- | --- | --- | --- | --- | --- | --- | --- | --- | --- | --- | --- | --- | --- | --- | --- | --- | --- |
|  | *petB-petD* | | | | | | *trnS2-trnG2* | | | | | | *atpH-atpI* | | | | | | | | | | | *ndhA* | | | | | | | | | | *petN-psbM* | | | | | | | | | | | |
|  | 76 | 132 | 497 | 525 | 543 | 594 | 999 | 1103 | 1107 | 1218 | 1251 | 1445 | 1631 | 1634 | 1730 | 1749 | 1777 | 1820 | 1867 | 1913 | 1971 | 2013 | 2051 | 2442 | 2543 | 2573 | 2658 | 2853 | 2856 | 2899 | 2904 | 2930 | 3017 | | 3112 | 3177 | 3198 | 3280 | 3345 | 3362 | 3388 | 3404 | 3450 | 3699 | 3740 |
| C1 | G | C | C | C | T | C | T | A | C | G | C | A | T | A | * | T | G | # | A | T | A | G | C | T | G | A | A | A | T | C | A | G | G | | T | A | T | G | A | C | C | A | T | T | A |
| C2 | G | C | C | C | T | C | T | A | C | G | C | A | T | A | * | T | G | # | A | T | A | G | C | T | A | A | A | A | T | C | A | G | G | | T | A | T | C | A | C | T | A | T | T | A |
| C3 | G | C | C | C | T | C | T | A | C | G | C | A | T | A | * | T | G | # | A | T | A | G | C | T | G | A | A | A | T | C | A | G | G | | T | A | T | C | A | C | C | A | T | T | A |
| C4 | G | C | C | C | T | C | T | A | C | G | C | A | T | A | * | T | G | # | A | T | A | G | C | T | G | A | A | A | T | T | C | G | G | | T | A | T | G | A | C | C | A | T | T | A |
| C5 | G | C | C | C | T | C | T | A | C | G | C | A | T | A | * | T | G | # | A | T | A | G | C | G | A | A | A | A | T | C | A | G | G | | T | A | T | C | A | C | T | A | T | T | A |
| C6 | G | C | C | C | G | C | T | A | C | G | C | A | T | A | * | T | G | # | A | T | A | G | C | T | G | A | A | A | T | C | A | G | G | | T | A | T | G | A | C | C | A | T | T | A |
| C7 | G | C | C | C | T | C | T | A | C | G | C | A | G | A | * | T | G | # | A | T | A | G | C | T | G | A | G | A | T | C | A | G | G | | T | A | T | C | A | C | C | A | T | T | A |
| C8 | A | C | C | C | T | C | T | G | C | G | C | A | T | A | * | T | G | & | A | T | A | G | T | T | G | A | A | A | T | C | A | T | G | | T | T | T | G | A | C | C | A | T | T | A |
| C9 | A | C | C | C | T | C | T | G | C | G | C | A | T | A | * | T | G | & | A | T | A | G | T | T | G | A | A | A | T | C | A | G | G | | T | T | T | G | A | C | C | A | T | T | A |
| C10 | G | C | C | C | T | C | T | A | C | G | C | A | T | A | * | T | G | # | A | T | A | G | C | T | G | A | A | A | T | C | C | G | G | | T | A | T | G | A | C | C | A | T | T | A |
| C11 | G | C | C | C | T | C | T | A | C | G | C | A | T | C | * | T | G | # | A | T | A | G | C | T | G | A | A | A | T | C | C | G | G | | T | A | T | G | A | C | C | A | T | T | A |
| C12 | A | C | C | C | T | C | T | G | C | A | C | C | T | A | * | T | G | & | A | T | A | G | T | T | G | A | A | A | T | C | A | G | G | | T | T | T | G | A | C | C | A | T | T | A |
| C13 | G | C | T | C | T | C | T | A | C | G | C | A | T | A | * | T | G | # | A | T | A | G | C | T | G | A | A | A | T | C | A | G | G | | T | A | T | G | A | C | C | A | G | T | A |
| C14 | G | C | C | C | T | T | T | A | C | G | C | A | T | A | * | T | G | # | A | T | A | G | C | T | G | A | A | A | G | C | A | G | G | | T | A | T | G | A | C | C | A | G | T | A |
| C15 | A | C | C | C | T | C | T | G | C | G | C | A | T | A | * | T | A | & | A | T | A | G | T | T | G | A | A | A | T | C | A | G | G | | T | T | T | G | A | T | C | A | T | T | A |
| C16 | G | C | C | C | T | T | T | G | C | G | C | A | T | A | * | T | G | # | A | T | A | G | C | T | G | A | A | A | G | C | A | G | G | | T | A | T | G | A | C | C | A | G | T | A |
| C17 | A | C | C | C | T | C | T | G | C | G | C | A | T | A | * | T | A | & | A | T | A | G | T | T | G | A | A | A | T | C | A | G | G | | T | A | T | G | A | C | C | A | G | T | A |
| C18 | G | C | C | C | T | C | T | A | C | G | C | C | T | A | * | T | G | # | A | T | A | G | C | T | G | A | A | A | T | C | A | G | G | | T | A | T | G | A | C | C | A | T | T | A |
| C19 | A | C | C | C | T | C | T | G | C | G | C | A | T | A | * | T | G | & | A | T | A | G | T | T | G | A | A | T | T | C | A | G | G | | T | T | T | G | A | C | C | A | T | T | A |
| C20 | A | C | C | C | T | C | T | G | C | G | C | A | T | A | * | T | G | & | A | T | A | G | T | T | G | A | A | A | T | C | A | G | G | | T | T | C | G | G | C | C | A | T | T | A |
| C21 | G | C | C | C | T | C | T | A | C | G | C | A | T | A | * | T | G | # | A | T | A | G | C | T | G | A | A | A | T | C | A | G | G | | T | A | T | G | A | C | C | C | T | T | A |
| C22 | G | C | C | C | T | C | T | A | C | G | C | A | T | A | * | T | G | # | A | T | A | G | C | T | G | A | A | A | T | C | A | G | G | | T | A | T | C | A | C | T | A | T | T | A |
| C23 | A | A | C | C | T | C | T | G | C | G | C | A | T | A | * | T | G | & | A | T | A | G | T | T | G | A | A | A | T | C | A | G | G | | T | T | T | G | A | C | C | A | T | T | A |
| C24 | G | C | C | C | T | C | T | A | C | G | C | A | T | A | * | G | G | & | A | T | A | G | C | T | G | G | A | A | T | C | A | G | G | | T | T | T | G | A | C | C | A | T | T | A |
| C25 | A | C | C | C | T | C | T | G | C | G | C | A | T | A | $ | T | G | & | A | T | A | G | T | T | G | A | A | A | T | C | A | G | G | | T | T | T | G | A | C | C | A | T | T | A |
| C26 | G | C | C | C | T | C | T | A | C | G | C | A | T | A | * | T | G | & | A | T | A | T | C | T | G | A | A | A | T | C | A | G | G | | T | T | T | G | A | C | C | A | T | T | A |
| C27 | A | C | C | C | T | C | T | G | C | G | C | A | T | A | * | T | G | & | A | C | A | G | T | T | G | A | A | A | T | C | A | G | G | | T | T | T | G | A | C | C | A | T | T | A |
| C28 | A | C | C | C | T | C | T | G | C | G | C | A | T | A | * | T | G | & | A | T | A | G | T | T | G | A | A | A | T | C | A | G | G | | T | T | T | G | G | C | C | A | T | T | A |
| C29 | A | C | C | C | T | C | T | G | C | G | C | A | T | A | * | T | A | & | A | T | A | G | T | T | G | A | A | A | T | C | A | G | G | | T | T | T | G | A | C | C | A | T | C | A |
| C30 | G | C | C | C | T | C | T | A | C | G | T | A | T | A | * | T | G | # | A | T | A | G | C | T | G | A | A | A | T | C | A | G | G | | T | A | T | G | A | C | C | A | T | T | A |
| C31 | G | C | C | C | G | C | T | A | C | G | C | A | T | A | * | T | G | # | A | T | A | G | C | T | G | A | A | A | T | C | A | G | A | | T | A | T | G | A | C | C | A | T | T | A |
| C32 | G | C | C | C | G | C | T | A | C | G | C | A | T | A | * | T | G | # | C | T | A | G | C | T | G | A | A | A | T | C | A | G | G | | T | A | T | G | A | C | C | A | T | T | A |
| C33 | G | C | C | C | T | C | C | A | C | G | C | A | T | A | * | T | G | # | A | T | A | G | C | T | G | A | A | A | T | C | A | G | G | | T | A | T | G | A | C | C | A | T | T | A |
| C34 | G | C | C | C | G | C | T | A | C | G | C | C | T | A | * | T | G | # | A | T | A | G | C | T | G | A | A | A | T | C | A | G | G | | T | A | T | G | A | C | C | A | T | T | A |
| C35 | G | C | C | T | G | C | T | A | C | G | C | A | T | A | * | T | G | # | A | T | A | G | C | T | G | A | A | A | T | C | A | G | G | | T | A | T | G | A | C | C | A | T | T | A |
| C36 | G | C | C | C | T | C | T | A | C | G | C | A | T | A | * | T | G | # | A | T | G | G | C | T | G | A | A | A | T | C | A | G | G | | T | A | T | G | A | C | C | A | T | T | A |
| C37 | G | C | C | C | T | C | T | A | C | G | C | A | T | A | * | T | G | # | A | T | A | G | C | T | G | A | A | A | T | C | A | G | G | | G | A | T | C | A | C | C | A | T | T | A |
| C38 | G | C | C | C | T | C | T | A | A | G | C | A | T | A | * | T | G | # | A | T | A | G | C | T | G | A | A | A | T | C | A | G | G | | T | A | T | G | A | C | C | A | T | T | A |
| C39 | G | C | C | C | T | C | T | A | C | G | C | A | T | A | * | T | G | & | A | T | A | G | C | T | G | A | A | A | T | C | A | G | G | | T | T | T | G | A | C | C | A | T | T | A |
| C40 | G | C | C | C | G | C | T | A | C | G | C | A | T | A | * | T | G | # | A | T | A | G | C | T | G | A | A | A | T | C | A | G | G | | T | A | T | G | A | C | C | A | T | T | C |
| C41 | G | C | C | C | T | C | T | G | C | G | C | A | T | A | * | T | G | # | A | T | A | G | C | T | G | A | A | A | T | C | A | G | G | | T | A | T | C | A | C | C | A | T | T | A |

Note: *, -------------------; $, TTACATTTCCTTGTTTCTA; #, ----------; &, ACAGTCATAA

**Supplementary Table S2** Genetic information and frequencies of chlorotypes and ribotypes in populations of *R. Soongarica.*

| Population  (code) | cpDNA | | | | |  | ITS | | | | |
| --- | --- | --- | --- | --- | --- | --- | --- | --- | --- | --- | --- |
|  | Chlorotypes(individual) | *s* | *h* | *H*_d_ | *π* |  | Ribotypes(individual) | *s* | *h* | *H*_d_ | *π* |
| ALS | C1(2) C2(1) C3(2) C4(2) C5(1) | 6 | 5 | 0.8929 | 0.0007 |  | R1(8) R2(8) | 3 | 2 | 0.5333 | 0.0024 |
| EJN | C2(7) C10(1) | 4 | 2 | 0.2500 | 0.0003 |  | R1(7) R2(8) R6(1) | 6 | 3 | 0.5917 | 0.0030 |
| HSW | C13(1)C14(2)C15(2) C16(1) C17(1) C18(1) | 11 | 6 | 0.9286 | 0.0014 |  | R4(7) R12(7) R13(1) R14(1) | 2 | 4 | 0.6500 | 0.0012 |
| HZ | C13(7) C21(1) | 3 | 2 | 0.2500 | 0.0002 |  | R4(1) R12(4) R20(2) R21(4) R22(1) R23(3) R24(1) | 7 | 7 | 0.8667 | 0.0035 |
| JT | C3(6) C6(1) C18(1) | 3 | 3 | 0.4643 | 0.0002 |  | R2(4) R4(4) R12(5) R26(3) | 3 | 4 | 0.7917 | 0.0019 |
| JYH | C1(1) C2(2) C10(4) C22(1) | 4 | 4 | 0.7500 | 0.0005 |  | R1(7) R4(7) R12(1) R27(1) | 5 | 4 | 0.6500 | 0.0036 |
| LHT | C1(7) C26(1) | 2 | 2 | 0.2500 | 0.2500 |  | R2(1) R3(1) R4(4) R12(4) R26(4) R40(1) R41(1) | 7 | 7 | 0.8500 | 0.0031 |
| MJW | C1(2) C6(2) C30(4) | 2 | 3 | 0.7143 | 0.0003 |  | R1(8) R4(8) | 4 | 2 | 0.5333 | 0.0032 |
| MQSWS | C1(3) C6(1) C31(3) C32(1) | 3 | 4 | 0.7857 | 0.0004 |  | R1(7) R4(7) R52(1) R53(1) | 6 | 4 | 0.6500 | 0.0039 |
| QYS | C1(2) C3(3) C33(1) C34(1) C35(1) | 5 | 5 | 0.8571 | 0.0005 |  | R1(1) R4(7) R25(6) R54(1) R55(1) | 16 | 5 | 0.7000 | 0.0079 |
| SSC | C3(1) C31 (1) C35(2) C36(4) | 5 | 4 | 0.7500 | 0.0005 |  | R1(7) R4(8) R56(1) | 4 | 3 | 0.5917 | 0.0032 |
| THCY | C1(2) C3(1) C37(3) C38(2) | 3 | 4 | 0.8214 | 0.0004 |  | R1(7) R2(7) R4(1) R58(1) | 4 | 4 | 0.6500 | 0.0028 |
| WD | C1(1) C2(4) C3(2)C39(1) | 4 | 4 | 0.7500 | 0.0005 |  | R1(8) R2(8) | 3 | 2 | 0.5333 | 0.0024 |
| WW | C1(1) C6(1) C34(1) C35(3) C40(2) | 4 | 5 | 0.8571 | 0.0004 |  | R1(8) R2(8) | 3 | 2 | 0.5333 | 0.0024 |
| YC | C1(1) C3(6) C41(1) | 2 | 3 | 0.4643 | 0.0001 |  | R1(8) R2(8) | 3 | 2 | 0.5333 | 0.0024 |
| ZY | C1(5) C6(1) C31(2) | 2 | 3 | 0.6071 | 0.0003 |  | R1(8) R59(8) | 3 | 2 | 0.5333 | 0.0024 |
| KS | C9(6) C24(1) C25(1) | 5 | 2 | 0.2500 | 0.0003 |  | R4(6) R18(6) R36(1) R37(1) R38(1) R39(1) | 19 | 6 | 0.7500 | 0.0079 |
| BC | C8(5) C9(3) | 1 | 2 | 0.5357 | 0.0001 |  | R4(8) R5(8) | 4 | 2 | 0.5333 | 0.0032 |
| MF | C28(8) | 0 | 1 | 0.0000 | 0.0000 |  | R4(6) R18(1) R43(1) R44(2) R45(1) R46(2) R47(1) R48(1) R49(1) | 8 | 9 | 0.8583 | 0.0055 |
| LP | C9(7) C27(1) | 1 | 2 | 0.2500 | 0.0001 |  | R4(8) R30(2) R42(6) | 6 | 3 | 0.6333 | 0.0048 |
| KEKZ | C9(3) C23(5) | 1 | 2 | 0.5357 | 0.0001 |  | R4(6) R18(1) R28(1) R29(1) R30(6) R31(1) | 8 | 6 | 0.7500 | 0.0051 |
| HT | C9(6) C19(1) C20(1) | 3 | 3 | 0.4643 | 0.0002 |  | R4(6) R15(2) R16(2) R17(2) R18(1) R19(3) | 7 | 6 | 0.8250 | 0.0051 |
| JQ | C1(1) C3(7) | 1 | 2 | 0.2500 | 0.0001 |  | R1(4) R2(8)R25(4) | 4 | 3 | 0.6667 | 0.0030 |
| AX | C1(1) C6(6) C7(1) | 4 | 3 | 0.4643 | 0.0003 |  | R1(8) R3(8) | 2 | 2 | 0.5333 | 0.0016 |
| SSG | C9(8) | 0 | 1 | 0.0000 | 0.0000 |  | R1(8) R57(8) | 3 | 2 | 0.5333 | 0.0024 |
| YW | C2(8) | 0 | 1 | 0.0000 | 0.0000 |  | R1(8) R4(8) | 4 | 2 | 0.5333 | 0.0032 |
| CDH | C2(5) C6(3) | 4 | 2 | 0.5357 | 0.0006 |  | R1(8) R3(8) | 2 | 2 | 0.5333 | 0.0016 |
| KLKH | C6(3) C9(5) | 5 | 2 | 0.5357 | 0.0007 |  | R2(6) R32(1) R33(1) R34(7) R35(1) | 5 | 5 | 0.7000 | 0.0030 |
| MHG | C1(2) C6(2) C29(4) | 7 | 3 | 0.7143 | 0.0010 |  | R2(7) R4(7) R50(1) R51(1) | 3 | 4 | 0.6500 | 0.0015 |
| SCK | C2(1) C6(5) C9(2) | 8 | 3 | 0.6071 | 0.0008 |  | R1(8) R2(8) | 3 | 2 | 0.5333 | 0.0024 |
| YQ | C2(4) C6(1) C9(2) C22(1) | 8 | 4 | 0.7500 | 0.0010 |  | R1(8) R4(8) | 4 | 2 | 0.5333 | 0.0032 |
| FK | C10(7) C11(1) | 1 | 2 | 0.2500 | 0.0001 |  | R7(5) R8(8) R9(3) | 7 | 3 | 0.6583 | 0.0055 |
| HSS | C12(8) | 0 | 1 | 0.0000 | 0.0000 |  | R10(8) R11(8) | 4 | 2 | 0.5333 | 0.0032 |
| SW | C10(8) | 0 | 1 | 0.0000 | 0.0000 |  | R8(8) R31(8) | 5 | 2 | 0.5333 | 0.0040 |
| All |  | 42 | 41 | 0.9210 | 0.0010 |  | 59 | 66 | 59 | 0.8643 | 0.0060 |

Note:*s*, number of segregating sites; *h*, number of haplotypes; *H*d, estimates of haplotype diversity; *π*, nucleotide diversity within populations.

**Supplementary Table S3** Estimates of genetic variation on ITS sequences.

| Group**s** | *H*_S_ | *H*_T_ | *G*_st_ | *N*_st_ | *V*_s_ | *v*_T_ |
| --- | --- | --- | --- | --- | --- | --- |
| TaD | 0.732(0.047) * | 0.820(0.025) * | 0.108(0.047) * | 0.165(0.044) * | 0.692(0.078) | 0.828(0.076) |
| QB | 0.590(0.035) * | 0.846(0.047) * | 0.303(0.019) * | 0.364(NS) | 0.545(0.238) | 0.857(0.357) |
| BJ-TD | 0.586(0.048) * | 0.790(0.040) * | 0.258(0.042) * | 0.265(0.059) | 0.581(0.076) | 0.790(0.076) |
| GuD | 0.575(0.041) * | 0.917(0.048) * | 0.373(0.091) | 0.532(0.239) | 0.455(0.168) | 0.973(0.060) |
| KD-GG | 0.567(0.033) * | 0.813(0.046) * | 0.303(NS) | 0.266(NS) | 0.591(0.121) | 0.805(0.156) |
| Total | 0.630(0.019) * | 0.873(0.022) * | 0.279(0.021)* | 0.386(0.034)* | 0.538(0.058) | 0.876(0.112) |

Note: *H*_S_, average gene diversity within populations; *H*_T_, total gene diversity; *G*st, interpopulation differentiation; *N*st, number of substitution types; *P* values are enclosed in the parenthesis; NS, no significant; *, *P* <0.05.

**Supplementary Table S4** List of the bioclimatic variables defining the ecological niche of *R. soongarica.*

| code | Bioclimatic variables | Percent contribution | Permutation importance |
| --- | --- | --- | --- |
| **Bio_19** | Precipitation of Coldest Quarter | **40.4** | **59.2** |
| **Bio_11** | Mean Temperature of Coldest Quarter | **23.1** | **23.8** |
| **Bio_13** | Precipitation of Wettest Month | 9.8 | 3.7 |
| **Bio_9** | Mean Temperature of Direst Quarter | 5.4 | 2.8 |
| **Bio_18** | Precipitation of Warmest Quarter | 4.7 | 2.8 |
| **Bio_4** | Temperature Seasonality (standard deviation *100) | 4.6 | 0.6 |
| **Bio_3** | Isothermality (BIO2/BIO7)(*100) | 3 | 1.1 |
| **Bio_15** | Precipitation Seasonality(Coefficient of Variation) | 2.6 | 1.1 |
| **Bio_8** | Mean Temperature of Wettest Quarter | 2.3 | 2.3 |
| **Bio_5** | Max Temperature of Warmest Month | 2 | 0.8 |
| **Bio_2** | Mean Diurnal Range(Mean of monthly (max temp-min temp) | 1.9 | 0.9 |
| **Bio_14** | Precipitation of Driest Month | 0.1 | 0.9 |

Note: Significant contributions over 10% are indicated in bold text.

**Supplementary Table S5** List of locality information for the populations of sampled *R. soongarica.*

| **Population**  **(code)** | **Location**  **(All in China)** | **Groups** | **Latitude (N)** | **Longitude (E)** | **Altitude(m)** |
| --- | --- | --- | --- | --- | --- |
| **ALS** | Alxa left Banner, IMG | BJ-TD | 38°35.998´ | 105°38.400´ | 1373 |
| **EJN** | Ejin Banner, IMG | BJ-TD | 42°0.846´ | 101°40.152´ | 914 |
| **HSW** | Haishiwan, Gansu | BJ-TD | 36°20.990´ | 102°52.968´ | 1768 |
| **HZ** | Huzhu, Qinghai | BJ-TD | 36°38.429´ | 101°52.614´ | 2639 |
| **JT** | Jingtai, Gansu | BJ-TD | 37°21.682´ | 104°8.178´ | 1611 |
| **JYH** | Juyanhai, IMG | BJ-TD | 42°15.066´ | 101°14.648´ | 909 |
| **LHT** | Laohutai, Gansu | BJ-TD | 36°16.132´ | 103°49.236´ | 1880 |
| **MJW** | Mengjiawan, Ningxia | BJ-TD | 37°26.117´ | 104°54.720´ | 1380 |
| **MQSWS** | Mingqin, Gansu | BJ-TD | 38°52.687´ | 103°20.040´ | 1373 |
| **QYS** | Quanyanshan, IMG | BJ-TD | 37°29.345´ | 105°33.648´ | 1201 |
| **SSC** | Shashichang, Ningxia | BJ-TD | 37°27.816´ | 104°25.998´ | 1872 |
| **THCY** | Tonghucaoyuan, IMG | BJ-TD | 37°35.860´ | 104°58.734´ | 1368 |
| **WD** | Wuda, IMG | BJ-TD | 39°32.338´ | 106°46.152´ | 1114 |
| **WW** | Wuwei, Gansu | BJ-TD | 39°19.644´ | 102°50.934´ | 1340 |
| **YC** | Yinchuan, Ningxia | BJ-TD | 38°7.368´ | 106°35.928´ | 1274 |
| **ZY** | Zhangye, Gansu | BJ-TD | 39°5.423´ | 100°28.350´ | 1459 |
| **KS** | Kashi, Sinkiang | TaD | 38°44.306´ | 76°14.227´ | 1496 |
| **BC** | Baicheng, Sinkiang | TaD | 41°50.183´ | 81°42.961´ | 1377 |
| **MF** | Mingfeng, Sinkiang | TaD | 36°50.125´ | 82°57.941´ | 1889 |
| **LP** | Luopu, Sinkiang | TaD | 36°50.605´ | 80°9.848´ | 1708 |
| **KEKZ** | Keerkezi, Sinkiang | TaD | 39°41.143´ | 75°22.126´ | 2090 |
| **HT** | Hetian, Sinkiang | TaD | 36°56.413' | 79°29.654' | 1840 |
| **JQ** | Jiuquan, Gansu | KD-GG | 40°12.882´ | 96°22.743´ | 1485 |
| **AX** | Anxi, Gansu | KD-GG | 40°32.294´ | 95°50.594´ | 1173 |
| **SSG** | Shashangou, Sinkiang | KD-GG | 39°39.858´ | 94°22.02´ | 2083 |
| **YW** | Yiwu,Sinkiang | KD-GG | 43°15.632´ | 94°50.705´ | 1609 |
| **CDH** | Chaidanhu, Qinghai | QB | 37°27.189´ | 95°30.994´ | 3182 |
| **KLKH** | Ke’lukehu, Qinghai | QB | 37°20.759´ | 96°57.988´ | 2838 |
| **MHG** | Mahuanggou, Qinghai | QB | 36°8.958´ | 100°0.972´ | 3029 |
| **SCK** | Sanchakou, Qinghai | QB | 37°27.346´ | 95°58.391´ | 3300 |
| **YQ** | Yuqia, Qinghai | QB | 38°1.667´ | 95°1.739´ | 3212 |
| **FK** | Fukang, Sinkiang | GuD | 44°18.874´ | 88°7.519´ | 554 |
| **HSS** | Huoshaoshan, Sinkiang | GuD | 44°51.683´ | 88°59.361´ | 873 |
| **SW** | Shawan, Sinkiang | GuD | 44°32.898´ | 85°44.188´ | 381 |

Note: *IM, Inner Mongolia; BJ-TD, Badan Jaran - Tengger desert; TaD, Taklimakan Desert; GuD, Gurbantunggut Desert; KD-GG, Kumtag desert and Gashun Gobi; QB, Qaidam basin.

**Supplementary Table S6** Twenty-three most variable regions in chloroplast genomes detected in this study.

| **Code** | **Region** | **name** | **Forward** | **Name** | **Reverse** |
| --- | --- | --- | --- | --- | --- |
| **1** | *petB-petD* | *petB-*f | CAATCCACTTTGACTCGTTTT | *petD-r* | GGTTCACCAATCATTGATGGTTC |
| **2** | *clpP* | *clpP-f* | GCTTGGGCTTCTCTTGCTGACAT | *clpP-r* | TCCTAATCAACCGACTTTATCGAG |
| **3** | *atpH-atpI* | *atpH-f* | AACAAAAGGATTCGCAAATAAAAG | *atpI-r* | AGTTGTTGTTCTTGTTTCTTTAGT |
| **4** | *trnH-psbA* | *trnH-f* | CGCGCATGGTGGATTCACAAATC | *psbA-r* | TGCATGGTTCCTTGGTAACTTC |
| **5** | *trnT-psbD* | *trnT-f* | GCCCTTTTAACTCAGTGGTAGAG | *psbD-r* | CCAAATAGGAACTGGCCAATC |
| **6** | *accD-psaI* | *accD-f* | GGTAAAAGAGTAATTGAACAAAC | *psaI-r* | GGAAATACTAAGCCCACTAAAGGCACA |
| **7** | *trnS2-trnG2* | *trnS2-f* | CGGTTTTCAAGACCGGAGCTATCAA | *trnG2-r* | CATAACCTTGAGGTCACGGGTTCAAAT |
| **8** | *atpH-atpI* | *atpH-f* | AACAAAAGGATTCGCAAATAAAAG | *atpI-r* | AGTTGTTGTTCTTGTTTCTTTAGT |
| **9** | *psbM-trnD* | *psbM-f* | TTTGACTGACTGTTTTTACGTA | *trnD-r* | CAGAGCACCGCCCTGTCAAG |
| **10** | *ndhC-trnV* | *ndhC-f* | AGACCATTCCAATGCCCCCTTTCGCC | *trnV-r* | GTTCGAGTCCGTATAGCCCTA |
| **11** | *ndhF* | *ndhF-f* | ACACCAACGCCATTCGTAATGCCATC | *ndhF-r* | AAGATGAAATTCTTAATGATAGTTGG |
| **12** | *petA-psbJ* | *petA-f* | GGATTTGGTCAGGGAGATGC | *psbJ-r* | ATGGCCGATACTACTGGAAGG |
| **13** | *psbE--petL* | *psbE-f* | ATCTACTAAATTCATCGAGTTGTTCC | *petL-r* | TATCTTGCTCAGACCAATAAATAGA |
| **14** | *ndhA* | *ndhA5’* | TCAACTATATCAACTGTACTTGAAC | *NdhA3’* | CGAGCTGCTGCTCAATCGAT |
| **15** | *rbcL-accD* | *rbcL-f* | TAGCTGCTGCTTGTGAGGTATGGA | *accD-r* | AAATACTAGGCCCACTAAAGG |
| **16** | *rpl32-trnL* | *rpl32-f* | GCGTATTCGTAAAAATATTTGGAA | *trnL-r* | TTCCTAAGAGCAGCGTGTCTACC |
| **17** | *rpoB-trnC* | *rpoB-f* | ACAAAATCCTTCAAATTGTATCTGA | *trnC-r* | TTTGTTAATCAGGCGACACCCGG |
| **18** | *rps16-trnQ* | *rps16-f* | TTTATCGGATCATAAAAACCCACT | *trnQ-r* | TGGGGCGTGGCCAAGCGGT |
| **19** | *petN-psbM* | *petN-f* | ATGGATATAGTAAGTCTCGCTTGG | *psbM-r* | ATGGAAGTAAATATTCTTGCAT |
| **20** | *trnK* | *trnK-f* | GGGACTCGAACCCGGAACTA | *trnK-r* | AGTACTCGGCTTTTAAGTGCG |
| **21** | *trnS^GCU^-trnG^GCC^* | *trnS^GCU^* | AACGGATTAGCAATCCGACGCTTTA | *trnG^GCC^* | CTTTTACCACTAAACTATACCCGC |
| **22** | *trnS^UGA^-**trnG^UCC^* | *trnS^UGA^* | CGGTTTTCAAGACCGGAGCTATCAA | *trnG^UCC^* | CATAACCTTGAGGTCACGGGTTCAAAT |
| **23** | *trnW-**psaJ* | *trnW-f* | TCTACCGAACTGAACTAAGAGCGC | *psaJ-r* | CGATTAATCTCTATCAATAGACCTGC |

**Supplementary Table S7** Characteristics of the primers and thermal programs for the five cpDNA fragments and nrITS.

| Region | Primer  （5’→3’） | sequence | amplified sequence length (bp) | Reference |
| --- | --- | --- | --- | --- |
| *pet*B-*pet*D | *pet*B | CAATCCACTTTGACTCGTTTT | 1030 | 2 |
|  | *pet*D | GGTTCACCAATCATTGATGGTTC |  |  |
| *trn*S2-*trn*G2 | t*rn*S2 | CGGTTTTCAAGACCGGAGCTATCAA | 1040 | 2 |
|  | *trn*G2 | CATAACCTTGAGGTCACGGGTTCAAAT |  |  |
| *atp*H-*atp*I | *atp*H | AACAAAAGGATTCGCAAATAAAAG | 940 | 2 |
|  | *atp*I | AGTTGTTGTTCTTGTTTCTTTAGT |  |  |
| *ndh*A | *ndh*A | TCAACTATATCAACTGTACTTGAAC | 940 | 2 |
|  | *Ndh*A | CGAGCTGCTGCTCAATCGAT |  |  |
| *pet*N-*psb*M | *pet*N | ATGGATATAGTAAGTCTCGCTTGG | 1020 | 2 |
|  | *psb*M | ATGGAAGTAAATATTCTTGCAT |  |  |
| *ITS*1-*ITS*4 | *ITS*1 | TCCGTAGGTGAACCTGCGG | 690 | 3, 4 |
|  | *ITS*4 | TCCTCCGCTTATTGATATGC |  |  |

**Supplementary Method S1**

DNA amplifications were performed in 20 μL reaction mixtures with high-fidelity Taq polymerase Plus PCR MasterMix (TIANGEN, Beijing, China), based on the thermal program consisting of initial denaturation at 95°C for 4 min followed by 34 cycles of 95°C for 30 s, 56°C of *pet*B-*pet*D, 58°C of *atp*H-*atp*I, *ndh*A, 59°C of *pet*N-*psb*M, 64°C of *trn*S2-*trn*G2 for 1 min, 72°C for 1 min and a final extension of 72°C for 7 min. For the amplification of the nuclear ITS fragment, the following program was used: 3 min at 95°C, 32 cycles of 30 s at 95°C, 30 s of annealing at 60°C, 1 min at 72°C, and a final extension step at 72°C for 10 min.

**Supplementary Method S2**

Tajima’s *D* and Fu’s *F*_S_ were calculated to acessed possible expansions^5, 6^. Due to an excess of rare new mutations, the values of *D* and *F*_S_ should be significant negative within a clade under the expansion hypothesis. 10,000 replicates were conducted to calculate the significance of the tests.  All of these demographic tests were performed using ARLEQUIN v.3.1^7^.

**Supplementary Method S3**

Only the homogenous haplotypes were regarded as the population colonization and expansion. We carefully separated the haplotypes based on the genealogical tree and only pooled the samples from the homogenous genetic resources for further estimations in BEAST. Moreover, Bayesian skyline plots (BSPs) were simulated in BEAST to profile the scenarios of the effective population size (*N*e) change for each group with parameter settings similar to those specified above for the divergence time estimates^8^. A sample of molecular sequences from each region was used to estimate a posterior distribution of regional effective population size without dependence on a pre-specified parametric model of demographic history.

**References:**

1. Rogers, A. R., Harpending, H. Population growth makes waves in the distribution of pairwise genetic differences. *Mol. Phylogenet. Evol.* **9**, 552-569 (1992).
2. Dong, W., Liu, J., Yu, J., Wang, L. & Zhou, S. Highly variable chloroplast markers for evaluating plant phylogeny at low taxonomic levels and for DNA barcoding. *PLoS One* **7**, e35071 (2012).
3. White, T. J., Bruns, T., Lee, S. & Taylor, J. W. *Amplification and Direct Sequencing of Fungal Ribosomal RNA Genes for Phylogenetics*. (Academic Press, New York, 1990).
4. Baum, D. A., Small, R. L. & Wendel, J. F. Biogeography and floral evolution of *baobabs* (Adansonia, Bombacaceae) as inferred from multiple data sets. *Syst. Biol.* **47**, 181-207 (1998).
5. Tajima, F. Statistical method for testing the neutral mutation hypothesis by DNA polymorphism. *Genetics* **123**, 585–595 (1989).
6. Fu, Y. X. Statistical tests of neutrality of mutations against population growth, hitchhiking and background selection. *Genetics* **147**, 915–925 (1997).
7. Excoffier, L., Laval, G., Schneider, S. Arlequin (version 3.0): an integrated software package for population genetics data analysis. *Evol. Bioinform. Online* **1**, 47-50 (2005).
8. Drummond, A. J., Rambaut, A., Shapiro, B. & Pybus, O. G. Bayesian coalescent inference of past population dynamics from molecular sequences. *Mol. Biol. Evol.* **22**, 1185-1192 (2005).
